# Biosynthesis of polyhydroxybutyrate by *Methylorubrum extorquens* DSM13060 is essential for intracellular colonization in plant endosymbiosis

**DOI:** 10.3389/fpls.2024.1302705

**Published:** 2024-02-02

**Authors:** Namrata Baruah, Roosa Haajanen, Mohammad Tanvir Rahman, Anna Maria Pirttilä, Janne J. Koskimäki

**Affiliations:** ^1^ Ecology and Genetics Research Unit, University of Oulu, Oulu, Finland; ^2^ Disease Networks, Faculty of Biochemistry and Molecular Medicine, University of Oulu, Oulu, Finland

**Keywords:** endophytes, endosymbiosis, intracellular, plant-microbe interaction, mutualism, oxidative stress, infection, colonization

## Abstract

*Methylorubrum extorquens* DSM13060 is an endosymbiont that lives in the cells of shoot tip meristems. The bacterium is methylotrophic and consumes plant-derived methanol for the production of polyhydroxybutyrate (PHB). The PHB provides protection against oxidative stress for both host and endosymbiont cells through its fragments, methyl-esterified 3-hydroxybutyrate (ME-3HB) oligomers. We evaluated the role of the genes involved in the production of ME-3HB oligomers in the host colonization by the endosymbiont *M. extorquens* DSM13060 through targeted genetic mutations. The strains with deletions in PHB synthase (*phaC*), PHB depolymerase (*phaZ1*), and a transcription factor (*phaR*) showed altered PHB granule characteristics, as *ΔphaC* had a significantly low number of granules, *ΔphaR* had a significantly increased number of granules, and *ΔphaZ1* had significantly large PHB granules in the bacterial cells. When the deletion strains were exposed to oxidative stress, the *ΔphaC* strain was sensitive to 10 mM HO· and 20 mM H_2_O_2_. The colonization of the host, Scots pine (*Pinus sylvestris* L.), by the deletion strains varied greatly. The deletion strain *ΔphaR* colonized the host mainly intercellularly, whereas the *ΔphaZ1* strain was a slightly poorer colonizer than the control. The deletion strain *ΔphaC* lacked the colonization potential, living mainly on the surfaces of the epidermis of pine roots and shoots in contrast to the control, which intracellularly colonized all pine tissues within the study period. In earlier studies, deletions within the PHB metabolic pathway have had a minor effect on plant colonization by rhizobia. We have previously shown the association between ME-3HB oligomers, produced by PhaC and PhaZ1, and the ability to alleviate host-generated oxidative stress during plant infection by the endosymbiont *M. extorquens* DSM13060. Our current results show that the low capacity for PHB synthesis leads to poor tolerance of oxidative stress and loss of colonization potential by the endosymbiont. Altogether, our findings demonstrate that the metabolism of PHB in *M. extorquens* DSM13060 is an important trait in the non-rhizobial endosymbiosis.

## Introduction

Plants carry communities of microbes inside all of their tissues, endophytes, which often have various positive effects on their hosts. Most endophytes, bacteria and fungi, promote plant growth and protect the host against pathogens and abiotic stress. The endophytes typically enter the plant from the rhizosphere from the points of lateral root emergence, cracks or wounds, or through stomata in stem and leaf surfaces (Hardoim et al., 2015). The majority of endophytes colonize in the intercellular spaces of roots (James et al., 1994; Ryan et al., 2008; Izumi, 2011; Hardoim et al., 2015), and a small group of endophytes can breach through the endodermis to the vascular tissues, from where they enter the shoots in the xylem transpiration flow (Compant et al., 2005). The endophytic bacteria are rarely observed inside cells, and when present, they usually colonize dead plant cells, or alternatively, the intracellular infection induces plant cell death (James and Olivares, 1998; White et al., 2014).

We have earlier identified an endophyte that intracellularly colonizes meristematic tissues of Scots pine trees (*Pinus sylvestris* L.) (Pirttilä et al., 2000; Koskimäki et al., 2015; Koskimäki et al., 2016). A specific feature of the facultative endosymbiont, *Methylorubrum extorquens* DSM13060, is the accumulation of bacterial cells near the plant nuclei (Koskimäki et al., 2015). *M. extorquens* DSM13060 significantly improves the growth and affects the stress tolerance of the host (Pohjanen et al., 2014; Koskimäki et al., 2022). The endosymbiont produces compounds that alleviate oxidative stress in both plant and bacterial cells, methyl-esterified 3-hydroxybutyrate oligomers (ME-3HB). These compounds are the break-down products of polyhydroxybutyrate (PHB), which the endosymbiont synthesizes from methanol produced by the plant (Koskimäki et al., 2016; Müller-Santos et al., 2021).

Besides the crucial role in global CO_2_ fixation, plants participate in the carbon budget by emissions of methanol. The methanol is produced in plant tissue from pectin by the methyl esterase enzyme, and nonenzymatically, from the methoxyl groups of pectin and lignin. Plants lack mechanisms for detoxifying methanol, which is mainly emitted through stomata (Nemecek-Marshall et al., 1995). Methylotrophs, such as *M. extorquens* DSM13060, living inside and on the plant tissues, consume the plant-produced methanol and transform it to non-toxic forms, such as PHB (Fall, 1996; Sy et al., 2005; Knief et al., 2008; Hardoim et al., 2015).

The ME-3HB oligomers produced by the endosymbiont *M. extorquens* DSM13060 from plant-derived methanol have potent antioxidative activity against hydroxyl radicals. They are the result of the action by the PhaZ1 depolymerase, which degrades PHB, as well as the PHB synthase PhaC, which synthesizes PHB (Koskimäki et al., 2016). The degradation and biosynthesis of PHB is coordinated by the transcription factor, PhaR, which is a master regulator of PHB metabolism (Nishihata et al., 2018). The oxidative burst induced in the plant tissues during microbial infection creates hydroxyl radicals (Mucha et al., 2012; Koskimäki et al., 2016). When pine seedlings are infected by *M. extorquens* DSM13060, the hydroxyl radicals accumulate at the infection site, and activation of the genes *phaC* and *phaZ1* along with degradation of PHB granules take place to suppress the oxidative stress by the ME-3HB oligomers (Koskimäki et al., 2016).

As *M. extorquens* DSM13060 is an endosymbiont that penetrates the host cells, and the ME-3HB oligomers have an antioxidative role in the infection of plant tissues (Koskimäki et al., 2016), we hypothesized that the capacity for PHB synthesis and degradation is crucial for colonization of the plant host. Therefore, we examined the importance of the enzymes involved in PHB metabolism in *M. extorquens* DSM13060 for plant colonization through targeted single gene mutations.

## Materials and methods

### Bacterial strains and cultivation

*Methylorubrum extorquens* DSM13060 [Genbank: AGJK00000000] was originally discovered from bud tissues of mature Scots pine in Oulu, Finland (65°0´ N; 25°30´ E) (Pirttilä et al., 2000; Koskimäki et al., 2015). The derivate fluorescent *M. extorquens* 13061 (Pohjanen et al., 2014) with two successive chromosomal GFP coding genes under constitutive promoter was used as the parent strain for generation of gene in-frame deletion mutants (Xi et al., 1999). Strains and plasmids used are described in **Table 1**. For isolation of genomic DNA, mutant construction, and inoculum preparation, *M. extorquens* 13061 and constructed mutants were grown by shaking at 28°C in ammonium mineral salts (AMS) medium (ATCC medium 784) supplemented with 1% (v/v) methanol (MeOH), 1% (w/v) sodium succinate, and appropriate antibiotics when required (Kanamycin 50 μg/ml). The AMS medium contained 0.66 g/l (NH_4_)_2_SO_4_, 1.0 g/l MgSO_4_-7H_2_O, and 0.015 g/l CaCl_2_-2H_2_O. In addition, after sterilization, 1.0 ml/l of AMS trace elements solution, 1 ml/l of stock A, and 20 ml/l of 1.0 M phosphate buffer were added. The AMS stock A contained 5.0 g/l Fe-NaEDTA and 2.0 g NaMoO_4_*2H_2_O. The AMS trace elements solution contained 0.5 g/l FeSO_4_*7H_2_O, 0.4 g/l ZnSO_4_*7H_2_O, 0.02 g/l MnSO_4_*H_2_O, 0.015 g/l H_3_BO_3_, 0.01 g/l NiCl_2_*6H_2_O, 0.25 g/l EDTA, 0.05 g/l CoCl_2_*6H_2_O, and 0.005 g/l of CuCl_2_*2H_2_O. The 1 M phosphate buffer consisted of 113.0 g/l K_2_HPO_4_ and 47.0 g/l KH_2_PO_4_.

**Table 1:** The strains and vectors used for the study.

Strains or plasmids	Description	Reference or source
*Escherichia coli*		
DH5α	*endA1 gyrSA96 hrdR17 (rK-mK-) supE44 recA1*; general host strain used for the transformation and propagation of plasmids	(Boyer and Roulland-dussoix, 1969)
SM10 λ pir	Donor strain (mobilizing strain) carrying the transfer genes of the broad host range IncP type plasmid RP4 integrated into its chromosome. Useful for mobilizing mobRP4 plasmids, Km^r^	BCCM ref. LMBP 3889
HB101-pRK2013	*E. coli* HB101 carrying a self-transmissible plasmid pRK2013 containing the broad host-range transfer system of RK2, Km^r^	(Figurski and Helinski, 1979)
*Methylorubrum extorquens*		
DSM13060	Wild type; a plant growth-promoting conifer endophyte	(Pirttilä et al., 2000)
13061	DSM13060 containing mTn5*gusA-pgfp21*-Km^r^ cassette (pFAJ1820)	(Pohjanen et al., 2014)
*Plasmids*		
pJET 1.2	Cloning vector for amplified PCR products; Ap^r^	Thermo Fisher Scientific
pT18mobsacB	Broad host-range cloning vector maintained in Gram-negative bacteria offering sucrose counter-selection allelic exchange, Tc^r^	(Wei et al., 2013)
pCM433	Broad host-range cloning vector maintained in Gram-negative bacteria offering bacteria allelic exchange, Chl^r^ and Ap^r^	(Marx, 2008)

*Escherichia coli* strains DH5α and SM10*λpir*, used for the mutant construction, were grown in Luria-Bertani (LB) medium (1% tryptone, 0.5% yeast-extract, 1% NaCl) at 37°C, supplemented either with 100 μg/ml ampicillin or with 10 μg/ml tetracycline.

### Seed sterilization, plant growth conditions, and bacterial inoculation

Seeds of Scots pine were prepared by heat treatment for 72 h at 55°C in the dark, followed by incubation in sterile water overnight at room temperature and surface sterilization with 3% calcium hypochlorite (w/v) for 20 min. Once the seeds were surface sterilized, they were rinsed three times with sterile water and grown on moist sterile vermiculite in glass jars. Germination took place after 7-10 days at 24± 3°C at 16/8 h photoperiod. For the inoculation, the bacteria were grown in AMS supplemented with 1% MeOH, and 1% sodium succinate, for 3-4 days. The bacterial cultures were diluted with sterile water to the density of 2.5×10^8^ CFU/ml, and 100 µl of the inoculum was pipetted onto each germinated pine seedling.

### Isolation of genomic DNA

Bacterial cells were cultured and grown to the late logarithmic phase (optical density at 600 nm (OD_600_) 0.8 – 1.0) and harvested by centrifugation at 6000×g for 5 minutes at 4° C. The bacterial pellet was ground in liquid nitrogen with mortar and pestle, and the genomic DNA was isolated using DNeasy Plant Mini kit according to the manufacturer’s instructions (Qiagen). The quality and concentration of DNA were analyzed by NanoDrop ND-1000 spectrophotometer (NanoDrop Technologies) and Qubit 2.0 fluorometer with dsDNA HS assay kit (Invitrogen).

### Construction of the *ΔphaR*, *ΔphaC*, and *ΔphaZ1* deletion strains

The genomic data were retrieved from the JGI genome portal (https://img.jgi.doe.gov/) and used to design primers specific for the genes *phaR*, *phaC*, and *phaZ1* (IMG gene accessions 2507326187, 2507325801, 2507328730 respectively; and primer sequences are listed in **Table 2**). *M. extorquens* 13061 was used for generation of targeted deletion mutants *ΔphaR*, *ΔphaC*, and *ΔphaZ1* according to the method described earlier (Schäfer et al., 1994). Briefly, 0.5 kb regions upstream (AB) and downstream (CD) of the target genes were amplified using primers with restriction sites at overlap sequences specified in **Table 2**. Upstream and downstream regions were joined (AB+CD) by overlap extension PCR (OE-PCR) using Phusion High-Fidelity DNA polymerase (Thermo Scientific). The amplified constructs were verified on agarose gel, purified, and cloned into the pJET 1.2 vector (CloneJET PCR Cloning Kit, Thermo Scientific). The plasmids were then electroporated (Gene Pulser Electroporation system, BioRad) into *E. coli* DH5α. Transformants were selected on LB agar plates supplemented with ampicillin (100 µg/ml) and incubated overnight at 37°C. The colonies were screened by colony PCR (DreamTaq polymerase, Thermo Scientific) specific for the deletion (**Tables 2**, **3**), and the constructs were confirmed by Sanger sequencing (Eurofins Genomics).

**Table 2:** The primers designed for constructing the mutants.

Deleted gene	Sequence	Primer name, Restriction site
*phaR*	TATTGAATTCTGCCAGCGATGTGAAGAAC	phaR-F, *EcoRI*
*phaR*	AATAAAGCTTAAGATCACCTCGTCCACGTC	phaR-R, *HindIII*
*phaC*	TATTACGCGTTTGGACTCGTTGCGGTTG	phaC-F, *MluI*
*phaC*	TATTGAGCTCACCCAGCAGGTAGGGAATG	phaC-R, *SacI*
*phaZ1*	TATTACGCGTTCGACGGGGAAGAACCAG	phaZ1-F, *MluI*
*phaZ1*	TATTGAGCTCAATCGCGGCCACTTCTTC	phaZ1-R, *SacI*

Restriction sites are underlined and sequences for overlap extension are italicized.

**Table 3:** The primers specific for the vectors used.

Plasmid	Sequences	Primer name
pJET1.2	CGACTCACTATAGGGAGAGCGGC	pJET1.2-F
pJET1.2	AAGAACATCGATTTTCCATGGCAG	pJET1.2-R
pT18mobsacB	TGTAAAACGACGGCCAGT	pT18mobsacB-F
pT18mobsacB	AACAGCTATGACATGA	pT18mobsacB-R
pCM433	TGTAAAACGACGGCCAGT	M13 (-21) F
pCM433	CAGGAAACAGCTATGAC	M13 R

The plasmid DNA was isolated using GeneJet plasmid miniprep kit (Thermo Scientific). The deletion constructs were restricted from the pJET 1.2 plasmids and cloned into the suicide vector, either pT18mobsacB (*ΔphaR*), or pCM433 (*ΔphaC*, *ΔphaZ1*). Transformation of the suicide vector(s) was carried out in *E. coli* SM10*λpir* by electroporation. The transformants were grown on LB agar containing either tetracyclin (10 µg/ml) or ampicillin (100 µg/ml) in the presence of 2% X-gal and IPTG (in the case of pT18mobsacB). The positive colonies were selected by blue-white screening and confirmed by colony-PCR (sequences listed in **Table 3**).

### Transposon mutagenesis and mutant screening

Conjugations of the deletion constructs *ΔphaR* and *ΔphaC* (pT18mobsacB) in *E. coli* SM10*λpir* were performed by triparental mating using the *E. coli* HB101::pRK2013 as a helper strain, and *M. extorquens* 13061 as a recipient, as described before (Heinze et al., 2018). For the *ΔphaZ1* construct in pCM433 plasmid, the conjugation was performed by biparental mating without the helper strain, as previously described (Choi et al., 2006; Marx, 2008). The individual cultures were grown until the OD_600_ of 0.8–1.0 was reached. The donor, recipient, and helper strains were mixed by resuspending after centrifugation at 3000×g, 4°C for 5 min. The mixed pellet was suspended in 1 ml of AMS supplemented with 1% MeOH, and 200 µl of the cell suspension was transferred on 0.22 μm filter discs and incubated on AMS agar supplemented with 1% MeOH for 48-72 hours at 28°C for conjugation. Dilutions were made thereafter by suspending the filters in liquid AMS plates supplemented with 1% MeOH, 1% sodium succinate, tetracycline (30 µg/ml) and rifampicin (50 μg/ml) for 48 hours at 28°C. Counter selections were made using 10% sucrose for lacZ selection. Correct transformants were identified by colony-PCR (**Table 3**).

### Bacterial growth curves

The *M. extorquens* 13061 and the deletion mutants *ΔphaR, ΔphaC*, and *ΔphaZ1* were grown in 30 ml of AMS liquid medium supplemented with 1% MeOH and 1% sodium succinate for 48-72 hours with shaking at 28°C until the cultures reached OD_600_ of 0.8-1.0. Precultures of each strain were adjusted to equal cell densities (10^8^ CFU/ml), and the OD_600_ was measured after 12 h, 24 h, 48 h, 72 h, and 96 h. The total number of viable cells were determined as colony-forming-units (CFU/ml) by plating dilution series on AMS with 1% MeOH and 1% sodium succinate and counting the colonies after 72 h. A minimum of two biological replicates with three technical repeats were measured for each time point.

### *In vitro* stress assays

In the heat shock stress assays, bacterial strains were grown in AMS supplemented with 1% MeOH, and 1% sodium succinate, reaching the logarithmic phase at 28°C for 2-3 days. The cultures were then transferred to an incubator for heat shock at 55°C for up to 60 mins under shaking at 150 rpm. The sampling was done at 0, 30, and 60 min, where 100 µl of the culture was harvested and serially diluted by 10-fold to count the CFU/ml. For the UV irradiation assay, the strains were transferred to UV radiation at 4.8 joules/m^2^ under a wavelength of 254 nm for 45 s. At 0 s (before UV irradiation) and at 45 s, 100 µl of the samples were harvested and plated on AMS with 1% MeOH, and 1% sodium succinate in 10-fold dilutions for the CFU counting. The hydroxyl radical-induced stress assays were performed for the deletion and control strains as described before (Koskimäki et al., 2016). Hydroxyl radicals (HO·) were generated by Fenton reaction using H_2_O_2_ and ferric iron (Fe^3+^) in a modified M9 minimal salts medium. The concentration of H_2_O_2_ was adjusted to 10 mM, 15 mM, and 20 mM, which was supplemented with and without FeCl_3_ at 0.26 mM, 0.39 mM, and 0.52 mM, respectively, to produce a stable flux of hydroxyl radicals (Moore et al., 2006). The assay was performed in 24-well culture plates in three biological replicates. The bacteria were cultured up to the late exponential phase and their final cell density was adjusted to OD_600_ of 0.8-1.0 equal to 10^8^ CFU/ml in each sample. The assay reactions were adjusted to a total volume of 2 ml and incubated at 28°C with orbital shaking at 150 rpm for 1 and 3 h. Five 10-fold serial dilutions (from 10^-1^ to 10^-5^ cells) were made per treatment, and 4-µl samples of each strain were pipetted on AMS with 1% MeOH, and 1% sodium succinate agar plates at every sampling interval. Up to five separate treatments were pipetted per Petri plate, including control without stress, control with H_2_O_2_ stress, with or without FeCl_3_, and the deletion strains with H_2_O_2_ stress, with, or without FeCl_3_. The culture plates were digitally imaged and analyzed after 24 and 48 h. All treatments were pipetted in triplicates and the experiment was repeated twice.

### Droplet digital PCR (ddPCR)

Samples from HO· growth stress on *M. extorquens* 13061 and the deletion strain *ΔphaZ1* were taken at 0 h, 1 h, and 3 h post-induction of stress (hpi). Cells in each sample were harvested by centrifugation at 5,000×g for 5 min at 4°C, treated with 10 volumes (v/v) of RNA shield stabilizing solution (Zymo Research), and incubated at 4°C overnight. RNA was extracted by *Quick*-RNA miniprep kit (Zymo Research), and the genomic DNA was removed with DNase I (Thermo Fisher Scientific). The quantity and quality of the total RNA was analyzed by NanoDrop ND-1000 spectrophotometer (Thermo Fisher Scientific) and agarose gel electrophoresis.

Complementary DNA (cDNA) was synthesized from 1 μg of total RNA with random hexamer primers using SuperScript III First-Strand Synthesis System (Thermo Fisher Scientific). The gene expression was analyzed with primers specific for *phaZ1* and *phaZ2*, designed by GenScript Real-time PCR primer designing software (Primer3, V2.0) (**Table 4**). The specificity of the primers was confirmed by melting curve analysis using the LightCycler 480 Real-Time PCR System (Roche Diagnostics), as previously (Koskimäki et al., 2016). Each 20-μl ddPCR reaction mixture consisted of 2×QX200 ddPCR EvaGreen supermix (Bio-Rad), 250 nM forward and reverse primers, and the cDNA sample (3 μl; 1:50 dilution). The reaction mixtures were loaded into a DG8 cartridge (Bio-Rad) with 70 μl of Droplet Generation Oil (Bio-Rad) to generate thousands of droplets using the QX200 Droplet Generator (Bio-Rad). Droplets of 40 μl from each well were transferred to a ddPCR 96-well PCR plate (Bio-Rad). PCR reactions were performed in a T100 Thermal Cycler (Bio-Rad) following the program: initial heating at 95°C for 5 min, 40 cycles of denaturation at 95°C for 30 s and annealing at 58°C for 1 min, followed by 4°C for 5 min, and final denaturation at 90°C for 5 min. The endpoint data collected using a Bio-Rad QX200 droplet reader was analyzed using the Bio-Rad QuantaSoft program. Fluorescence measurements were recorded in the appropriate channel in the reader. The reader counted the number of droplets containing the target sequence (positive) and droplets without the target (negative). Each positive droplet was assigned a value of one and negative droplets were assigned a value of zero. A Poisson correction was applied by the QuantaSoft program so that the mean number of target sequences per partition could be estimated. The results from samples with unusually low amplitude shifts (relative to other samples) were discarded and necessary repetitions were made. The experiment contained three biological replicates, and controls were analyzed with technical repeats.

**Table 4:** The primers used for the Droplet Digital PCR.

Gene	Sequence	Primer name
*phaZ1*	AGAGGGTGGTCTGGGAAC	ddPCR_PhaZ1-Fw
*phaZ1*	CGGTGATGAAGACCTGATGG	ddPCR_PhaZ1-Rv
*phaZ2*	ATCGAGACCTTCTACGACGA	ddPCR_phaZ2-Fw
*phaZ2*	CCTTCCACCGTCATCAGG	ddPCR_phaZ2-Rv

### Transmission electron microscopy (TEM)

Bacterial strains were grown at 28°C for 4-5 days with shaking in M9 minimal salts medium (Obruca et al., 2010; Koskimäki et al., 2016) supplemented 0.5% sodium succinate and 1% MeOH until OD_600_ ≥1.0. The bacterial cultures were then centrifuged at 5000×g for 3 min and washed once with 1×PBS (pH 7.4). The pelleted cells were first fixed in 1% glutaraldehyde and 4% formaldehyde in 0.1M phosphate buffer, and then post-fixed with 1% osmium tetroxide in acetone and embedded in Epon LX112 (Ladd Research Industries Inc.). Thin sections were cut with an ultramicrotome (Leica UC6), stained with uranyl acetate and lead citrate, and examined with a Tecnai G2 Spirit 120 kV transmission electron microscope. Images were captured by a Quemesa CCD camera (Olympus Soft Imaging Solutions GMBH).

A total of five biological replicates of *ΔphaR, ΔphaC*, and *ΔphaZ1* and the control *M. extorquens* 13061 were analyzed for cell number, PHB granule distribution, and the granular area. The components were measured in the imaging and processing platform ImageJ (Lam et al., 2021). The number of cells per biological replicate and their PHB granule distribution was measured with the automatic cell counter plugin. The area of the PHB granules was measured with the manual exclusion of background coupled with appropriate settings of scale length and width to ensure an unbiased universal approach.

### Confocal laser scanning microscopy (CLSM)

The seedlings were analyzed at 60 and 120 dpi (days-post-inoculation). A total of 10-12 seedlings/sampling interval/strain was analyzed. The experiment consisted of 3 biological replicates conducted over the period of a year. Roots and shoots were cut into 2-3 mm pieces and fixed in 4% paraformaldehyde (w/v), 0.1% glutaraldehyde (v/v), 20% glycerol (v/v), and 0.1 M sodium phosphate buffer (pH 7.4) at 4°C under vacuum. The root samples were fixed for 4 h and the shoots were fixed for 8 h, followed by overnight incubation at 4°C. The fixed tissues were cut into 30-40 µm sections with a cryomicrotome (Reichert-Jung 2800 Frigocut with 2040 microtome) and mounted on microscopy slides with Vectashield antifade mounting medium with DAPI (Vector Laboratories). The pine tissue sections were studied with CLSM (LSM 5 Pascal; Carl Zeiss) using Plan-Neofluor 40×/1.3 and Plan-Apochromat 63×/1.4 oil objectives. The GFP fluorophore was excited at a wavelength of 488 nm by an argon ion laser, and emissions were detected using a 505-530 nm band-pass filter. The background autofluorescence of the plant tissues was detected using a 670 nm long-pass filter. The projections of the channels were analyzed and merged using the Zeiss LSM Image Browser (ver. 4.2.0.121) and Zeiss ZEN ver. 2.5 software (Blue edition; Carl Zeiss Microscopy GmbH). Representatives from 50-60 CLSM images were selected for each strain for the figure panels.

### Light microscopy for hydroxyl radical detection

At 60 dpi, the roots of seedlings inoculated with water or *M. extorquens* DSM13060 were cut into segments of 3 mm and fixed with Metacarn (60% methanol (v/v), 30% chloroform (v/v), 10% glacial acetic acid (v/v)) for 15 h at +4°C. For detection of potential sites of hydroxyl radical production in the pine tissue, a double staining of the Fenton reagents, H_2_O_2_ and iron (Fe^2+^ or Fe^3+^) was performed (Liu et al., 2007; Mucha et al., 2012). The root tissues were re-hydrated through an ethanol-water series, after which the samples were washed two times in sterile distilled water. Then, the root tissues were incubated in 7% (w/v) potassium ferrocyanide for 24 h for Fe^3+^ detection. Alternatively, for the detection of Fe^2+^, the samples were treated with 7% (w/v) potassium ferricyanide in aqueous 3% (v/v) HCl for 48 h. For the detection of H_2_O_2_, the samples were incubated for 75 min in 1 mg/ml 3,3’-diaminobenzidine (pH 3.8). The root samples were then dissected to sections of 20-25 µm thickness using the cryomicrotome and mounted on microscopy slides. The samples were examined with a light microscope (LSM 5 Pascal) and Plan-Apochromat 63x/1.4 and Plan-Neofluor 40x/1.3 objectives.

### Statistical analyses

The data from the analysis of the PHB granules was collected in Excel and statistically analyzed using the Student’s *t-*tests at the significance level of *p*<0.05. The normal distribution of ddPCR data was determined using the Shapiro-Wilk’s test for normality, and equality of variances was analyzed by the Levene’s test. For data that was not normally distributed, the Kruskal-Wallis test was used. For all normally distributed datasets with equal variances, a one-way analysis of variance (ANOVA) was performed. For the datasets that showed statistical significance, Tukey’s test was performed. The box plot for ddPCR data was constructed in RStudio, version 2022.07.2 using packages ggpubr and ggplot2.

## Results

### Characterization of the deletion strains *in vitro*

#### Cell and PHB granule morphology

Transmission Electron Microscopy (TEM) was performed to evaluate the characteristics of PHB granules in the deletion strains. The strains were grown in the presence of minimal requirements of nitrogen and high carbon content to facilitating maximal PHB granule growth, and they showed differences in their pigmentation and turbidity (**Supplementary Figure 1**). Whereas the control and *ΔphaR* grew normally with a pink coloration of the cells, *ΔphaC* showed an opaque color with clumps formed in the growth medium, and *ΔphaZ1* grew with a more intense pink shade and higher turbidity (**Supplementary Figure 1**).

**Figure 1:**
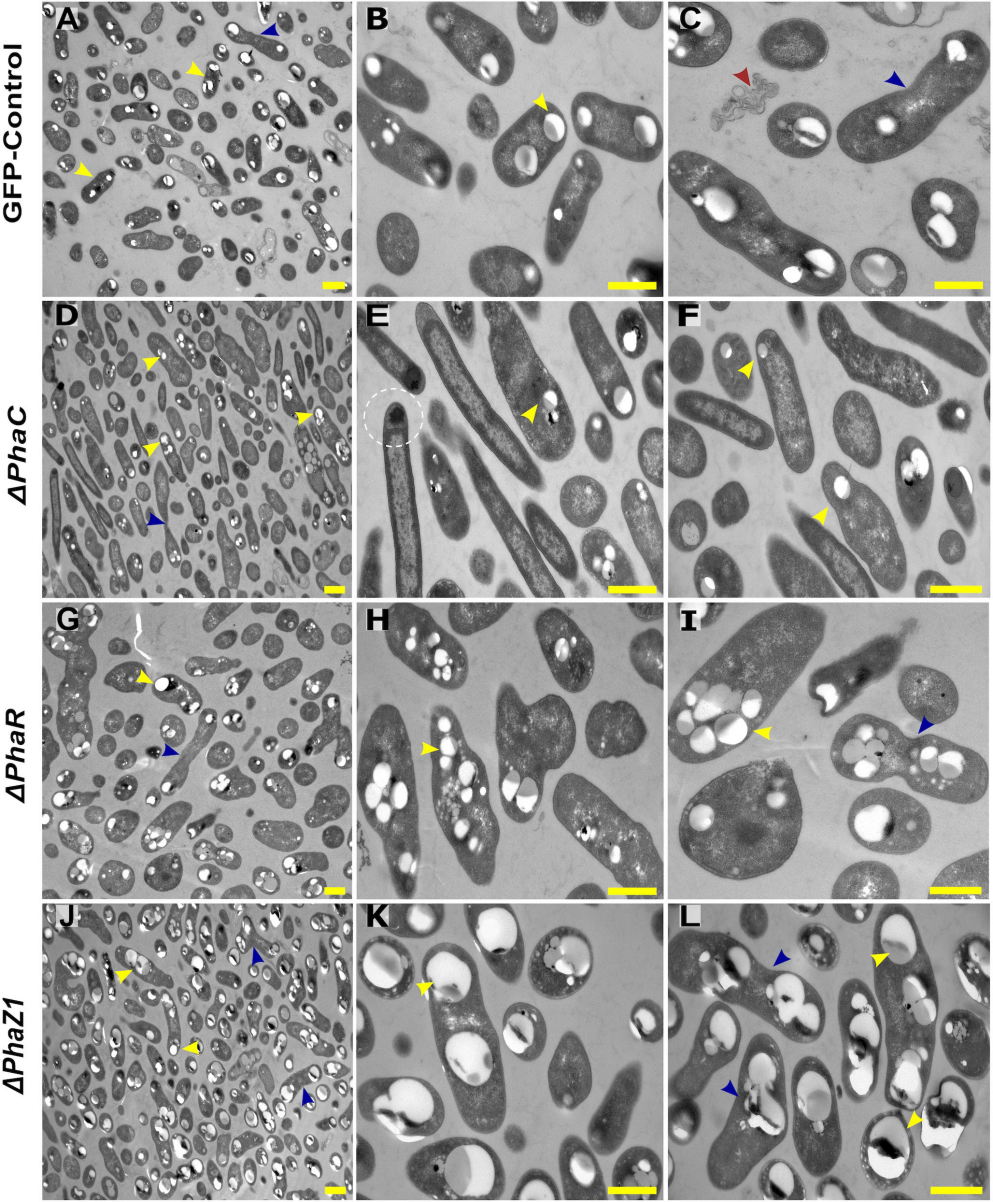
TEM images of PHB granules in the deletion mutant strains, *ΔphaC, ΔphaR, ΔphaZ1*, and control. **(A)** Cells of the control with a yellow arrowhead pointing the PHB granules, **(B, C)** 5× magnification of the control. The blue arrow shows a dividing cell, and the red arrow shows dead cellular accumulates. **(D)** *ΔphaC*, where the cells are irregularly shaped with a low number of small granules (yellow arrowhead) or the absence of granules. **(E, F)** A magnification showing mediation elements (circled), a single granule at the polar end of the cell (yellow arrowhead). **(G)** In *ΔphaR*, cells were irregular with numerous PHB granules per cell. **(H, I)** A magnification, where multiple granules are being distributed to the daughter cells (blue arrowhead). **(J)** *ΔphaZ1* cells had an increase in the volume of the granules. **(K, L)** A magnification showing enlarged masses of PHB granules (yellow arrowhead). The division of granules (blue arrowhead) was non-uniform. Scale bars, 1 μm.

**Figure 2:**
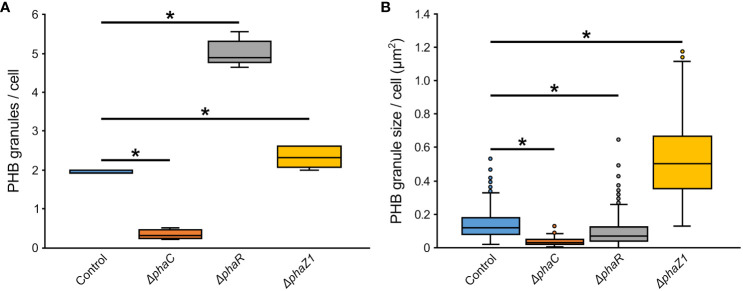
The number and size of PHB granules in *ΔphaR, ΔphaC*, and *ΔphaZ1* and control. **(A)** The number of PHB granules per cell of bacteria; **(B)** The size of PHB granules per cell in µm^2^. The analysis was made with 5 biological replicates (TEM images), and the values were quantified from the TEM images using the program ImageJ. The minimum and maximum values are denoted by whiskers; and the median is denoted by the black line inside each box, respectively. *=p<0.05 (Student’s *t*-test).

The control *M. extorquens* 13061 showed normal PHB granule size and distribution, having approximately two granules in each rod-shaped cell, and an even distribution of granules to daughter cells (**Figures 1A–C**; **2A, B**). The *ΔphaC* had an abnormal cell shape, being typically elongated (**Figures 1D–F**). The number of granules was significantly lower than in the control and most cells lacked granules (**Figures 1E, F**; **2A**). The size of the granule, when present, was also significantly smaller than in the control (**Figure 2B**), and the granules were unevenly transferred to the daughter cells (**Figure 1F**). A dark-stained mediation element (Tian et al., 2005a; Tian et al., 2005b) resembling polyphosphate granules (Quelas et al., 2013) was present in the polar segment of the *ΔphaC* cells (**Figure 1E**). In *ΔphaR*, the cells had an irregular shape accompanied by a significantly increased number of granules (4-6 granules/cell) (**Figures 1G–I**; **2A, B**). The volume of the granules was significantly smaller than in the control strain (**Figure 2B**), and the granules were unevenly distributed within the cytoplasm and to the daughter cells during cell division (**Figure 1I**). The cells of *ΔphaZ1* carried significantly larger granules compared to the control (**Figures 1J–L**; **2B**). Typically, there were 2-3 granules per cell (**Figures 1K**; **2A**).

#### Bacterial growth curves and stress tolerance

**Figure 3:**
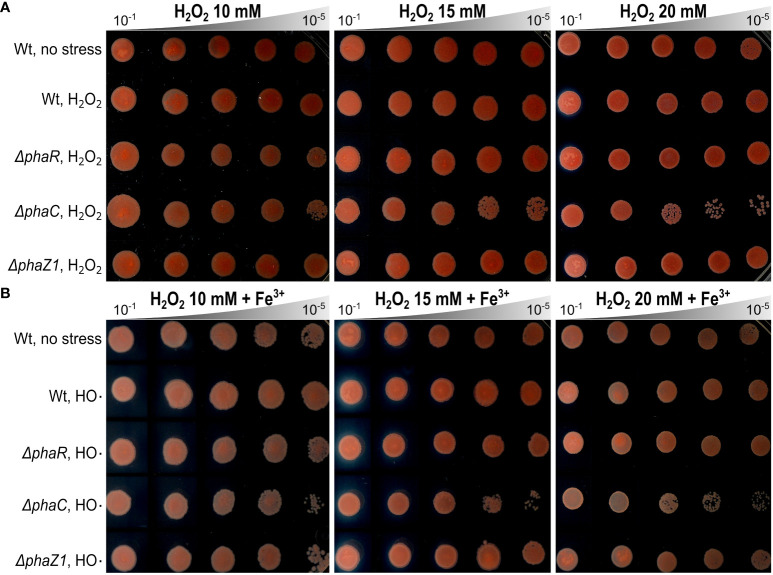
Oxidative stress assays. **(A)** Effect of hydrogen peroxide (H_2_O_2_) on the growth of *ΔphaR, ΔphaC, ΔphaZ1*, and the control 13061. **(B)** Effect of hydroxyl radicals (HO·), generated by Fenton reaction using H_2_O_2_ (as above) supplemented with FeCl_3_ (Fe^3+^). Data are representative of ten-fold serial dilutions of bacterial cells (10^-1^ to 10^-5^, gray triangle) from three independent experiments. Separately scanned images were combined, and the contrast adjustment was applied equally across the images. Wt refers to *M. extorquens* 13061.

The stress tolerance of the deletion strains *ΔphaR, ΔphaC*, and *ΔphaZ1* was tested based on their growth curves in normal growth conditions and under elevated temperature and UV irradiation. In the normal growth conditions, the deletion strains followed a similar growth pattern as the control (**Supplementary Figure 2A**). When a heat shock was applied, no difference in growth between the control and the deletion strains was seen. However, after 60 min of the heat shock, *ΔphaR*, *ΔphaZ1*, and the control ceased their growth, whereas *ΔphaC* survived with a few colonies growing (**Supplementary Figure 2B**). When the UV stress was applied, the strains showed decreased growth 45 s after UV irradiation with no significant differences between strains (**Supplementary Figure 2C**). When the hypersensitivity of the deletion strains to oxidative stress was investigated, the growth of *ΔphaR* and *ΔphaZ1* was unaffected in the presence of H_2_O_2_ and HO·, similar to the control. However, *ΔphaC* showed sensitivity to H_2_O_2_ at 20 mM, and to HO· already at 10 mM concentration (**Figure 3**).

#### Effect of HO· stress on gene expression

**Figure 4:**
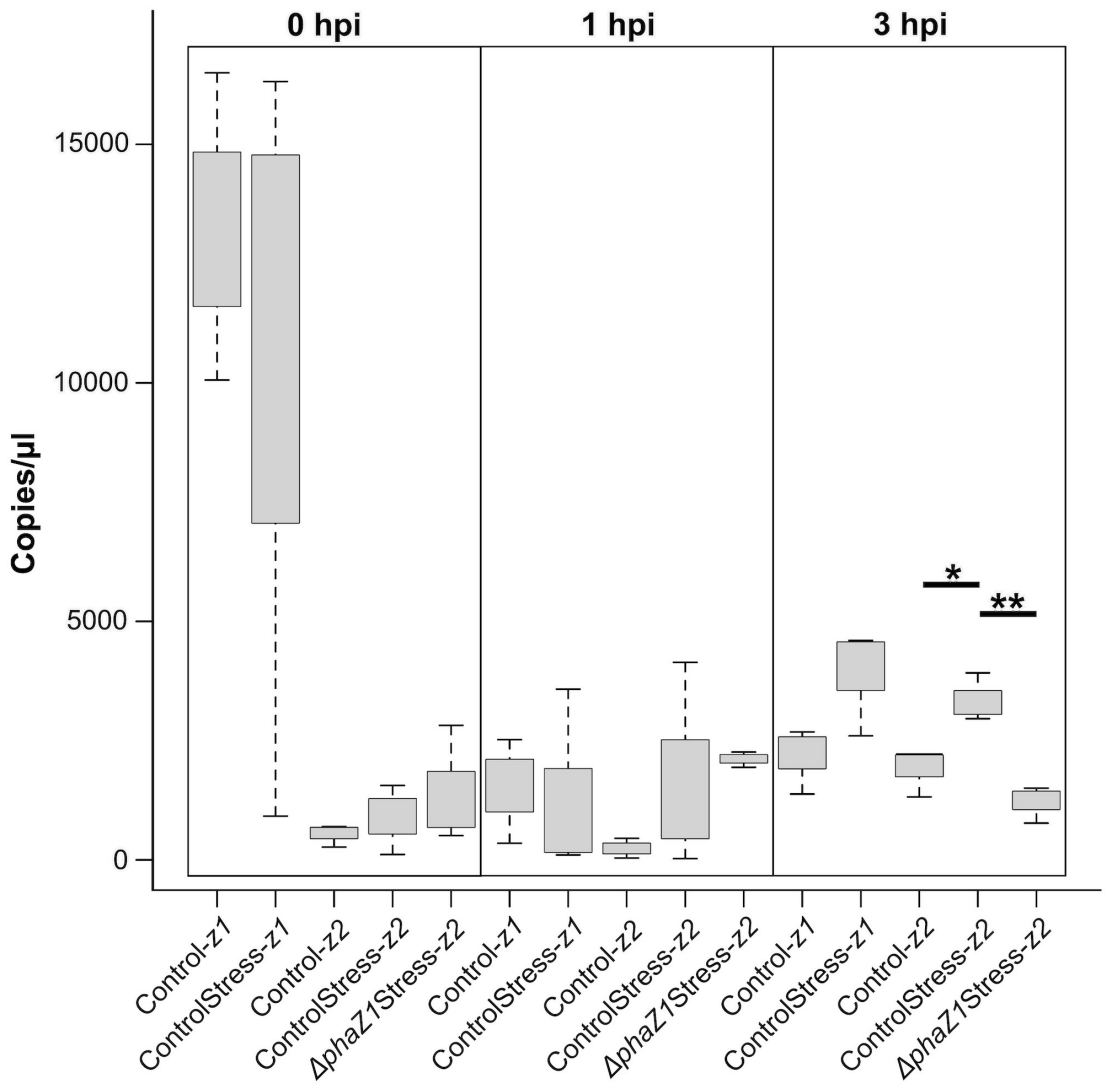
Gene expression of *phaZ1* and *phaZ2* under HO· stress in the control and *ΔphaZ1*. The average of three biological replicates at 0, 1 and 3 hpi, maximum and minimum values are denoted by whiskers and the median by the black line inside each box. The data was analyzed by one-way ANOVA and Tukey’s test, *=p<0.05, **=p<0.01.

To study whether another depolymerase gene of *M. extorquens* DSM13060, *phaZ2*, compensates for the loss of *phaZ1* in the deletion strain, the expression of *phaZ1* and *phaZ2* was studied by ddPCR in comparison to the control under HO· stress (15 mM H_2_O_2_, supplemented with 0.39 mM FeCl_3_). The expression of *phaZ2* gene was slightly but not statistically significantly induced in the *ΔphaZ1* strain at one hour post-induction (hpi) of stress compared to the control (**Figure 4**). However, at 3 hpi, the gene *phaZ2* had a significantly higher expression in the control than in *ΔphaZ1* (p<0.01) (**Figure 4**). The *phaZ1* gene had a high expression in the control in the beginning of the experiment and under stress after 3 hours, but the differences were not statistically significant (**Figure 4**).

### Colonization of plant tissue by the deletion strains

**Figure 5:**
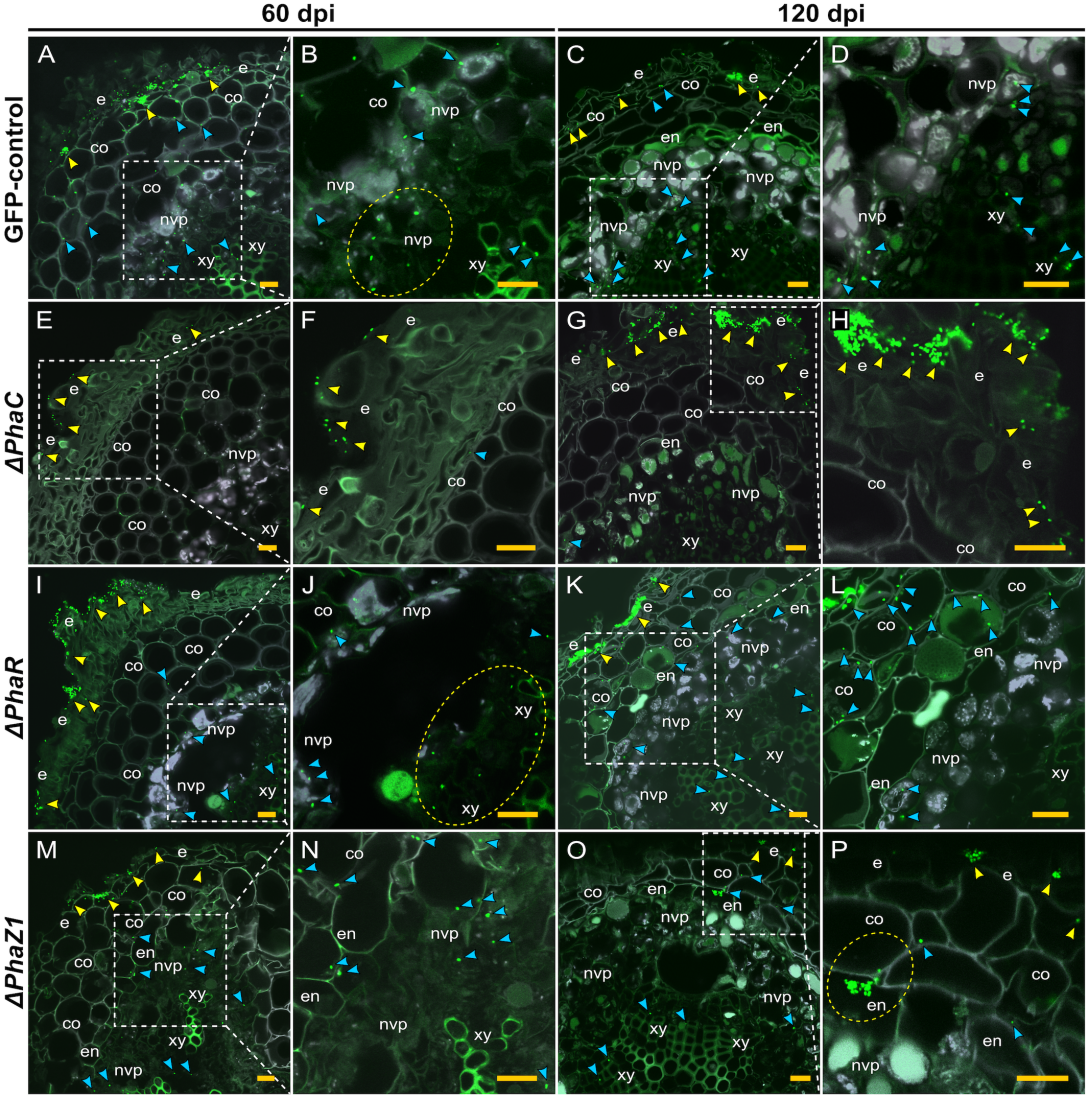
CLSM of pine roots colonized by the strains *ΔphaC, ΔphaR*, *ΔphaZ1*, and the control, *M. extorquens* 13061, 60 **(A, B, E, F, I, J, M, N)** and 120 **(C, D, G, H, K, L, O, P)** dpi. Bacterial cells labeled with a fluorescent GFP reporter under the control of a constitutive promoter are visualized in bright green, whereas the structures of plant tissue are visible mainly in grey color with the exception of lignified tissues of the endodermis and xylem, and cells of non-vascular parenchyma. Representatives of approximately 50-60 CLSM images are shown for each strain. **(A–D)** A cross-section of root inoculated with the control. Arrowheads indicate bacterial infection pockets (yellow) in the epidermis, and single bacterial cells (blue) in the plant cortical cells and **(B)** non-vascular parenchyma (encircled in the magnification). **(C)** Arrowheads show control bacteria (blue) colonizing further in the cells of the cortex and vascular tissue, and fewer cells are found in the infection pockets at the epidermis (yellow). **(D)** A magnification of **(C)**. **(E–H)** A cross-section of root inoculated with *ΔphaC*. **(E)** A low number of single *ΔphaC* cells (yellow arrowhead) is visible outside of the epidermis and, in the magnification **(F)**, a sporadic *ΔphaC* cell (blue) is found in the cortex. **(G)** The interior of pine root rarely hosts *ΔphaC* cells (blue arrowhead), but the quantity of *ΔphaC* cells outside of the epidermis has increased (yellow arrowhead), magnified in **(H)**. **(I–L)** A cross-section of root inoculated with *ΔphaR*. **(I)** Yellow arrowheads indicate numerous bacterial cells outside of the epidermis, and a number of *ΔphaR*. cells (blue) have reached the xylem (encircled), magnified in **(J)**. **(K)** Large infection pockets (yellow arrowhead) are present in the epidermis, and numerous *ΔphaR* cells (blue) are present between the cortical cells, magnified in **(L)**. **(M–P)** A cross-section of root inoculated with *ΔphaZ1*. **(M)** Small infection pockets (yellow arrowhead) are present in the epidermis, and several *ΔphaZ1* cells (blue) have colonized the cortex and vascular tissues, magnified in **(N)**. **(O)** Few *ΔphaZ1* cells are visible in the epidermis (yellow arrowhead), and a number of cells (blue) have reached the xylem tissue. **(P)** A magnification of **(O)**, where an infection pocket is formed by *ΔphaZ1* in the endodermis (encircled), and single *ΔphaZ1* cells (blue) are found in the cortex and xylem. Co, cortex; e, epidermis; en, endodermis; nvp, non-vascular parenchyma; xy, xylem. Dashed square=magnified area. Scale bars, 5 μm. Original CLSM images are shown as **Supplementary Material 2**.

When confocal laser scanning microscopy (CLSM) was performed on the strains *ΔphaC*, *ΔphaR*, and *ΔphaZ1*, we observed differences in the infection, colonization, and the intracellular establishment in the plant tissues by the bacteria. The control *M. extorquens* 13061 abundantly colonized the epidermal layer, the cortex (**Figure 5A**), and in the parenchymal cells of vascular tissue (**Figure 5B**) of pine roots at 60 days post-inoculation (dpi), as observed before (Koskimäki et al., 2015). At 120 dpi, the control had progressively advanced towards the vascular bundles (**Figure 5C**), where numerous cells were present in the xylem vessels (**Figure 5D**). In contrast, cells of the *ΔphaC* strain were unable to colonize the roots as rigorously (**Figures 5E–H**). At 60 dpi, single cells, instead of the microcolonies observed for the control, were found on the epidermis (**Figure 5E**). Sporadically, a cell or two were observed in the cortex, and no cells were observed in the endodermis or the xylem layers (**Figure 5F**). At 120 dpi, the *ΔphaC* strain formed large microcolonies and infection pockets on and in the epidermis (**Figures 5G, H**), but the number of bacteria was equally low in the cortex as at 60 dpi (**Figure 5H**) and still completely negligible in the endodermis and xylem (**Figure 5G**).

The deletion strains *ΔphaR* and *ΔphaZ1* had more similar patterns of pine root colonization as the control (**Figures 5I–P**). The cells of *ΔphaR* formed smaller infection pockets than the control in the epidermis at 60 dpi (**Figure 5I**), and individual cells were found in the xylem (**Figure 5J**). At 120 dpi, the *ΔphaR* had formed dense infection pockets in the epidermis (**Figure 5K**), and several singular cells were present in the intercellular spaces of cortical cells (**Figure 5L**). Although *ΔphaZ1* showed roughly a similar colonization pattern as the control within 60 days, the density of cells inside the tissues was lower (**Figures 5M, N**). There were infection pockets formed in the epidermis less frequently than in the control (**Figure 5M**). At 120 dpi, the *ΔphaZ1* cells had reached the xylem vessels (**Figure 5O**), and small infection pockets were visible in the epidermis, similar to the control (**Figure 5P**).

**Figure 6:**
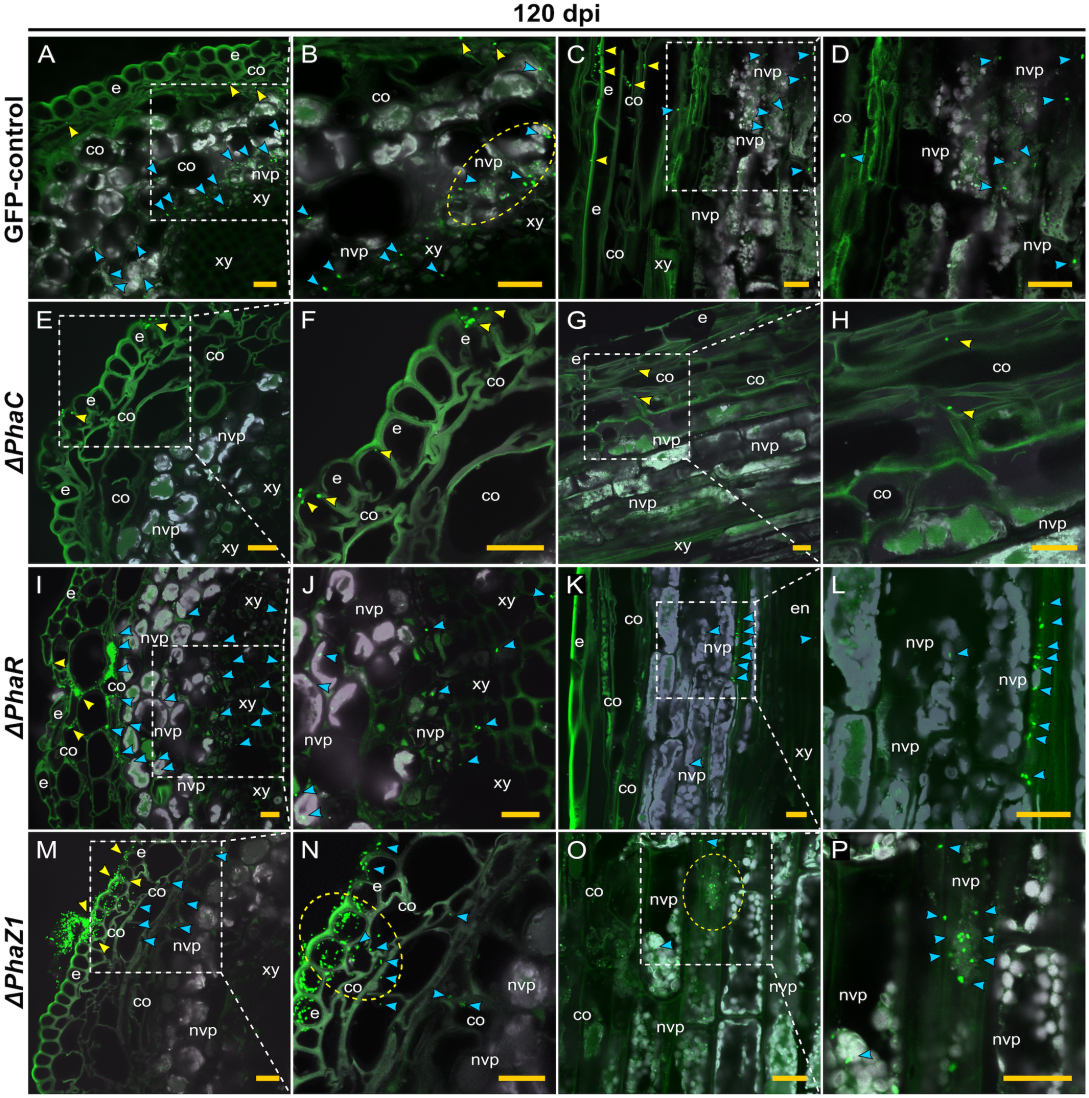
CLSM of pine shoots colonized by the strains *ΔphaC, ΔphaR*, *ΔphaZ1* and control, *M. extorquens* 13061, 120 dpi. Bacterial cells labeled with a fluorescent GFP reporter under the control of a constitutive promoter are visualized in bright green, whereas the structures of plant tissue are visible mainly in grey color with the exception of cuticula and lignified tissues of the epidermis. Representatives of 50-60 CLSM images are shown for each strain. Cross **(A, B)** and lateral **(C, D)** sections of pine shoot inoculated with the control. **(A)** Single control cells are found in the cortex (yellow arrowheads), and clusters of bacteria (blue) are visible in the non-vascular parenchyma, magnified in **(B)**. The bacterial cells of control (encircled) have aggregated around the plant non-vascular parenchyma. **(C)** Single bacterial cells of the control are visible inside the cortical cells (yellow arrowhead) and bacterial clusters are found in the cells of non-vascular parenchyma (blue), magnified in **(D)**. Cross **(E, F)** and lateral **(G, H)** sections of the shoot inoculated with the *ΔphaC* strain. **(E)** A low number of *ΔphaC* cells (yellow arrowhead) is visible in and on the epidermal cells, magnified in **(F)**. **(G)** Sporadic *ΔphaC* cells (yellow arrowhead) are present in the cortex, magnified in **(H)**. Cross **(I, J)** and lateral **(K, L)** sections of the shoot inoculated with the *ΔphaR* strain. **(I)** Infection pockets and individual cells of *ΔphaR* (yellow arrowhead) are visible between cortical cells, and a number of *ΔphaR* cells (blue) have reached the non-vascular parenchyma, magnified in **(J)**. **(K)** Blue arrowheads point out the mainly intercellular location of the *ΔphaR* cells, magnified in **(L)**. Cross **(M, N)** and lateral **(O, P)** sections of shoot inoculated with the *ΔphaZ1* strain. **(M)** The *ΔphaZ1* cells (yellow arrowhead) have breached the epidermis in numbers and colonize further (blue) the cells of cortex. **(N)** A magnification of **(M)**, where a cluster of *ΔphaZ1* cells penetrating through plant epidermis is encircled. **(O)** The *ΔphaZ1* cells have reached the non-vascular parenchyma (blue arrowhead), and several *ΔphaZ1*cells are aggregating around the plant nucleus (encircled), magnified in **(P)**. Co, cortex; e, epidermis; en, endodermis; nvp, non-vascular parenchyma; xy, xylem. Dashed square=magnified area. Scale bars, 5 μm. Original CLSM images are shown as **Supplementary Material 2**.

The colonization of pine shoots by *ΔphaR, ΔphaC*, and *ΔphaZ1* followed roughly the same patterns as in roots (**Figure 6**). However, the colonization of *ΔphaC* and *ΔphaZ1* was slower compared to the control, as they were not present in the samples of 60 dpi, hence the data are not shown. At 120 dpi, the control colonized throughout the shoot system (**Figures 6A–D**). The bacteria were present in the epidermis, the cortex, and in the vascular tissues (**Figures 6A, C**). The *ΔphaC* strain was found at very small quantities in the shoots at 120 dpi (**Figures 6E–H**). There were a few clusters of bacteria on the epidermal tissues (**Figures 6E, F**), and occasionally one or two individual cells were observed in the cortex (**Figures 6G, H**). The *ΔphaR* strain was present in the epidermis and cortex (**Figures 6I–L**), mainly intercellularly (**Figures 6I, L**). Individual bacterial cells were detected in the non-vascular parenchyma and xylem (**Figures 6J, L**). The *ΔphaZ1* strain showed an equal colonization in the shoots as the control (**Figures 6M–P**). The bacterial cells actively penetrated from microcolonies through the epidermal layer (**Figures 6M, N**) and accumulated in the non-vascular parenchyma cells (**Figures 6O, P**).

**Figure 7:**
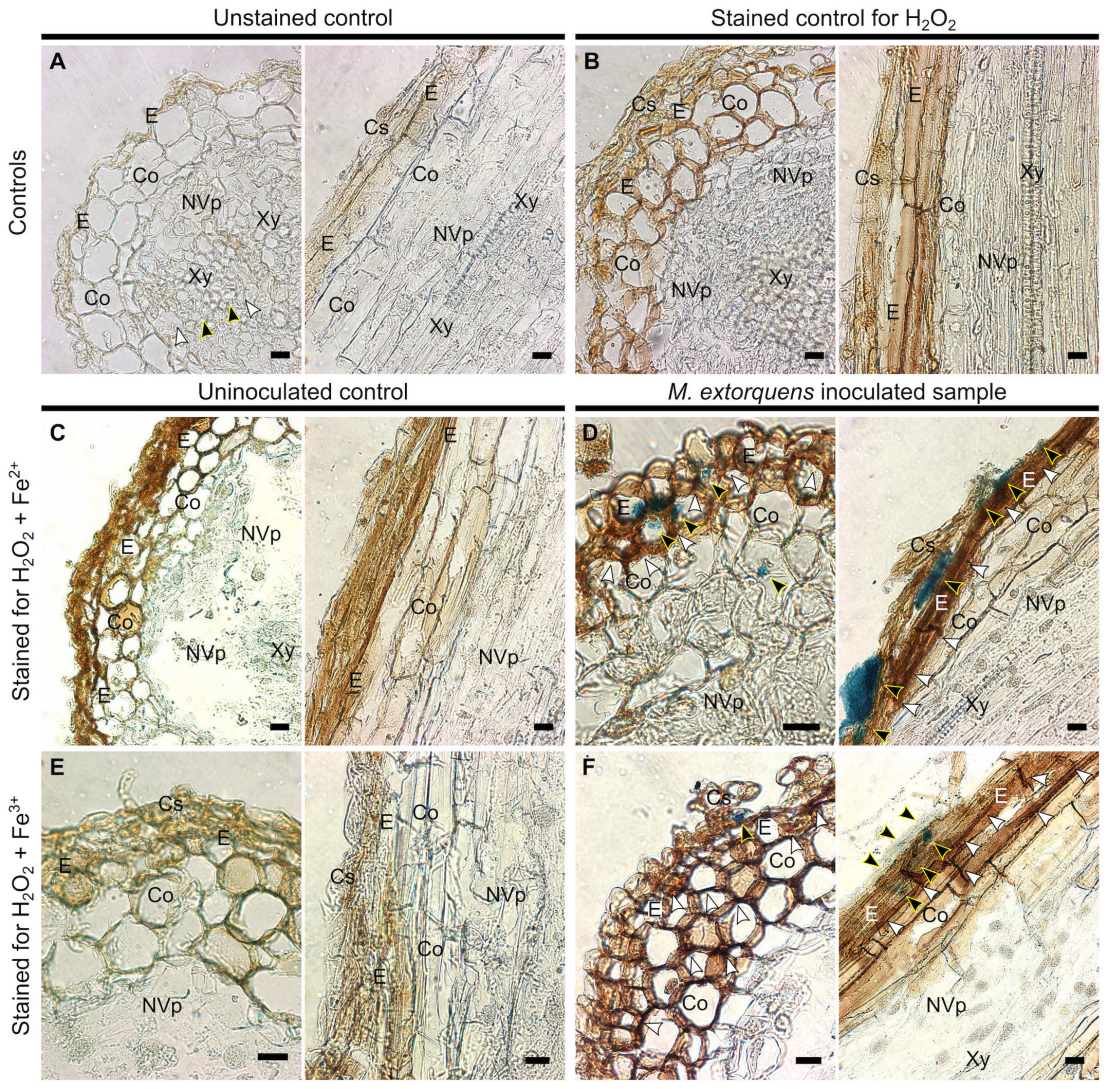
Light microscopy of Scots pine roots without inoculation (controls, **A-C, E**) or 60 dpi with *M. extorquens* DSM13060 (samples, **D, F**) stained for detection of H_2_O_2_ (brown), Fe^2+^, or Fe^3+^ (blue). **(A)** Cross and lateral sections of pine root of unstained control **(B)** and control stained for H_2_O_2_ **(C)** or Fe^2+^ and H_2_O_2_. **(D)** Cross and lateral sections of root inoculated with *M. extorquens* showing local deposits of Fe^2+^ in cortex and epidermis (black arrowheads) and local accumulation of H_2_O_2_ (white arrowheads) predominantly in the epidermis. **(E)** Cross and lateral sections of pine root of control stained for Fe^3+^ and H_2_O_2_. **(F)** Cross and lateral sections of inoculated root stained for Fe^3+^ and H_2_O_2_ showing colocalization of Fe^3+^ (black arrowheads) and H_2_O_2_ in cells of epidermis and cortex; black arrowheads indicate Fe^2+^ and Fe^3+^; white arrowheads indicate H_2_O_2_. Cs, cylindrical sheath; Co, cortex; E, epidermis; En, endoderm; NVp, nonvascular parenchyma; Xy, xylem. Scale bars, 20 μm.

To evaluate the presence of hydroxyl radicals in the pine tissue during infection, the Fenton reagents, H_2_O_2_ and iron (Fe ^2+^ and Fe^3+^), were localized in the pine root tissues at 60 dpi (**Figure 7**). The quantities of H_2_O_2_ and iron were low and uniformly distributed throughout the tissues of water-inoculated roots (**Figures 7A–C, E**). In the root tissues of *M. extorquens* DSM13060-inoculated seedlings, H_2_O_2_ and Fe^2+^ accumulated at specific sites of epidermis and cortex (**Figure 7D**), indicating the occurrence of oxidative burst and generation of hydroxyl radicals. The Fe^3+^ was co-localized with H_2_O_2_ to the same tissues as Fe^2+^, in the epidermal or cortical cells of the root tissue (**Figure 7F**).

## Discussion

Our previous results have shown that during the infection of the plant host, the endosymbiont *M. extorquens* DSM13060 utilizes polyhydroxybutyrate-derived compounds, ME-3HB oligomers, for protection against host-generated oxidative stress (Koskimäki et al., 2016). Therefore, we hypothesized that the deletion of the genes responsible for PHB synthesis and degradation, *phaC* and *phaZ1*, respectively, could affect the colonization of the host. We assumed that another gene potentially affecting the host colonization is *phaR*, which is a transcription factor that plays a key role in coordinating the other PHB-associated genes (Nishihata et al., 2018).

When the phenotypes of the deletion strains *ΔphaR, ΔphaC*, and *ΔphaZ1* of the endosymbiont *M. extorquens* DSM13060 were studied, their cellular morphologies varied to a degree. The cells of *ΔphaC* had very few or no PHB granules, which is in line with previous findings. For example, in *Azorhizobium caulinodans*, *ΔphaC* was unable to produce PHB granules, resulting in decreased growth rates and the loss of capacity for nitrogen fixation in the host (Crang et al., 2021). Although the draft genome of *M. extorquens* DSM13060 harbors only one known PHB synthase gene (Koskimäki et al., 2015), *ΔphaC* was able to occasionally produce low numbers of small PHB granules, which suggests that an additional copy exists within the unsequenced region. The cells of *ΔphaR* had numerous small PHB granules in the polar regions of the cells, and the cell shape was irregular. Deletion of *phaR* in the closely related *M. extorquens* AM1 has resulted in slow growth in the culture medium and a subsequent decrease in PHB synthesis (Korotkova et al., 2002), but the morphology of the cells has not been previously analyzed. Similarly, although the *phaZ* genes have often been deleted to enhance accumulation of medium chain-length polyhydroxyalkanoates in many bacterial species (Cai et al., 2009), the morphology of the deletion mutants has rarely been studied. The *ΔphaZ1* of *M. extorquens* DSM13060 accumulated PHB granules significantly greater in size and higher in number than the control granules, occasionally encompassing almost the entire volume of the cell. The *phaZ1* is an intracellular depolymerase with homology to the *phaZ2* of *R. eutropha* (York et al., 2003). Similar to *M. extorquens* DSM13060, the deletion of the *phaZ2* of *R. eutropha* leads to an increased granule volume and a higher number of granules per cell than in the wild type (Brigham et al., 2012).

When the deletion strains *ΔphaC, ΔphaR*, and *ΔphaZ1* were subjected to abiotic stress, similar growth patterns were observed, except that *ΔphaC* survived the heat shock in contrast to *ΔphaR* and *ΔphaZ1*, which arrested their growth. The better survival of *ΔphaC* under heat stress has been explained by the fact that hydroxybutyrate, the monomer of PHB, accumulates in the mutant and protects proteins from aggregation (Soto et al., 2012; Alves et al., 2020). However, in the oxidative stress assay, the deletion strain *ΔphaC* exhibited reduced growth rates compared to the control strain under 20 mM H_2_O_2_, and 10 mM HO·. In contrast, no significant differences between the control, *ΔphaR*, and *ΔphaZ1* were observed in the oxidative stress tolerance. To analyze if another *phaZ* gene, *phaZ2* of *M. extorquens* DSM13060 (Koskimäki et al., 2016) compensates for the loss of *phaZ1*, we studied the gene expression of *phaZ1* and *phaZ2* under oxidative stress. However, there was no significant difference in the *phaZ2* gene expression between *ΔphaZ1* and the control one hour after the HO· stress application, and after three hours, the *phaZ2* expression was significantly the highest in the stressed control. This suggests that *phaZ1* is the main gene responsible for PHB degradation in *M. extorquens* DSM13060 under oxidative stress. Generation of multiple deletions of *phaZ* genes would complete our understanding of the role of PHB depolymerases in stress tolerance of *M. extorquens* DSM13060.

Our previous results (Koskimäki et al., 2015; Koskimäki et al., 2016) have shown that the endosymbiont *M. extorquens* DSM13060 invades the host plant by similar mechanisms as the stem-colonizing rhizobia. It enters the plant actively through the epidermis in roots and through epidermis or stomatal apertures in stem, and forms infection pockets upon entry. The bacterial cells invade intracellularly through the endoderm further into the vascular tissues, leading to systemic colonization of the *in vitro*-grown Scots pine seedling within three months (Koskimäki et al., manuscript). In the current study, the control strain showed a successful colonization of pine seedlings as described. However, the deletion strains, *ΔphaC, ΔphaR*, and *ΔphaZ1* demonstrated mixed levels of colonization in the pine roots and shoots compared to the control. The *ΔphaZ1* of *M. extorquens* DSM13060 was a poorer colonizer in pine seedlings than the control, as no bacteria were identified in the shoots after 60 days. The infection and colonization process of *ΔphaR* differed from the control. Whereas the same tissues were colonized by *ΔphaR* as by the control, at an equal quantity and pace, the colonization was mainly intercellular instead of intracellular. The PhaR is responsible for regulating PHB biogenesis (Nishihata et al., 2018), and therefore we propose that the coordination of PHB degradation upon host cell infection was disrupted by the *phaR* deletion in *M. extorquens* DSM13060, preventing intracellular entry. The pine colonization by the deletion strain *ΔphaC* was strikingly different from the control. The *ΔphaC* basically lacked the potential for plant colonization in both root and shoot tissues. Majority of the bacteria resided epiphytically on the epidermis, and only sporadic single cells were observed in the deeper tissues.

In earlier studies, the biosynthesis and degradation of PHB has mainly been studied from the point of view of carbon metabolism (Trainer and Charles, 2006) due to the traditionally recognized role of PHB as a bacterial carbon storage (Lemoigne, 1926). Therefore, the plant colonization potential of the deletion strains has been linked with the availability of host carbon and the stringency of the plant defense system (Müller-Santos et al., 2021). In general, the extent of host colonization by the deletion strains has greatly depended on the type of symbionts and their hosts. For example, in the endophyte *Azospirillum brasilense*, an impaired *phaC* gene resulted in reduced colonization on both wheat and maize plants (Kadouri et al., 2003a; Kadouri et al., 2003b). In the rhizobial strain *Sinorhizobium meliloti*, the *phaC* deletion mutant was able to colonize and nodulate the host plant *Medicago* sp., but the symbiotic properties, such as nodule development and the number of nodules formed, were reduced (Aneja et al., 2005). In contrast, the *ΔphaR* deletion strain of *Bradyrhizobium diazoefficiens* was more competitive than the wild type for nodule formation, and performed better in symbiosis with soybean plants, although the bacteria were unable to produce PHB granules (Quelas et al., 2016).

Considering intracellular plant colonization, deletions within the PHB metabolism pathway have a minor effect on the colonization by rhizobia, such as *ΔphaC* or *ΔphaZ* of *S. meliloti, Rhizobium etli*, or *R. leguminosarum* bv. *phaseoli* (Trainer and Charles, 2006; Trainer et al., 2010) and *ΔphaR* of *B. diazoefficiens* (Quelas et al., 2016). In our study on *M. extorquens* DSM13060, the deletion strains *ΔphaZ1* and *ΔphaR* had an impaired capacity for plant colonization and, specifically, the deletion of the *phaC* gene had a drastic effect on the colonization potential by this intracellular symbiont. Besides losing the capacity for plant colonization, the *ΔphaC* deletion strain had a poor tolerance of oxidative stress. We have previously linked the generation of ME-3HB oligomers from PHB by the PhaC and PhaZ1 enzymes with the ability to alleviate oxidative stress during host infection by *M. extorquens* DSM13060 (Koskimäki et al., 2016). In our current study, the colonization by *ΔphaC* was halted at the epidermis, which also accumulated the hydroxyl-radical generating Fenton reagents H_2_O_2_, Fe^2+^, and Fe^3+^ upon bacterial infection. Based on our results, the PHB metabolism is more important in endosymbiotic interactions than in rhizobial ones, potentially due to the lack of a finely tuned cellular recognition machinery that exists in the rhizobial symbiosis. Therefore, endosymbiotic strains, such as *M. extorquens* DSM13060, need to alleviate the host-induced oxidative stress through PHB metabolism for a successful entry in the plant host. Altogether, our findings demonstrate that the metabolism of PHB is an important trait in the intracellular plant-microbe interaction due to the high antioxidative power of this molecule (Koskimäki et al., 2016).

## Data availability statement

The original contributions presented in the study are included in the article/**Supplementary Material**. Further inquiries can be directed to the corresponding author.

## Author contributions

NB: Software, Writing – review & editing, Data curation, Formal analysis, Investigation, Methodology, Visualization, Writing – original draft. RH: Formal analysis, Investigation, Methodology, Data curation, Writing – original draft. MR: Methodology, Software, Writing – review & editing. AMP: Conceptualization, Funding acquisition, Project administration, Resources, Software, Supervision, Validation, Writing – review & editing. JJK: Methodology, Software, Writing – review & editing, Conceptualization, Formal analysis, Investigation, Resources, Supervision, Visualization.
