# Supplementary material for: Biosynthesis of polyhydroxybutyrate by Methylorubrum extorquens DSM13060 is essential for intracellular colonization in plant endosymbiosis
Source: Front Plant Sci. 2024 Feb 2;15:1302705. doi: 10.3389/fpls.2024.1302705 (PMC10883064; doi:10.3389/fpls.2024.1302705)
Supplement: Supplementary file 1 [file DataSheet_1.pdf]

SUPPLEMENTARY FILES

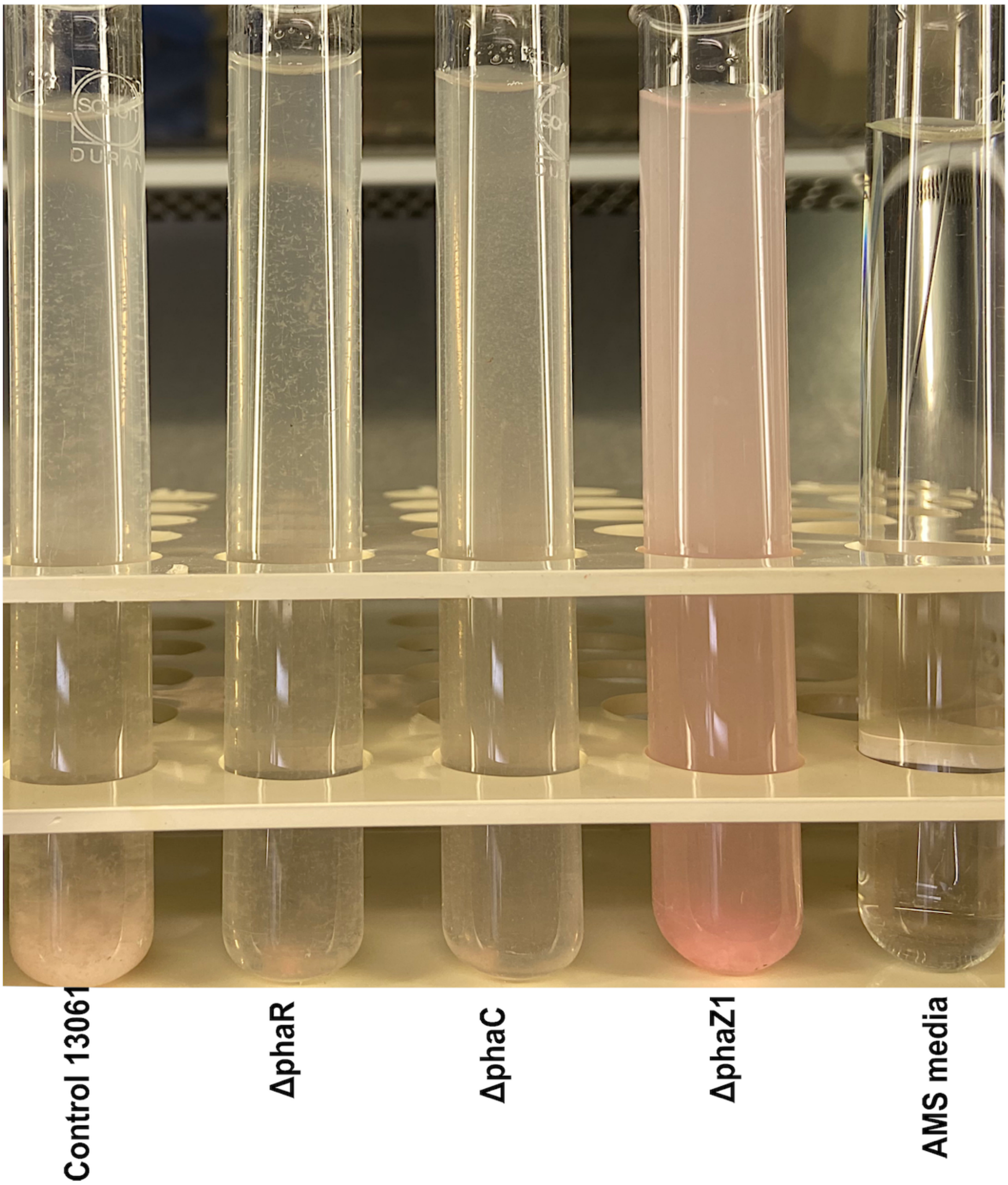

Figure S1. Cultures of the control *M. extorquens* 13061 and the deletion strains *ΔphaR*, *ΔphaC*, and *ΔphaZ1,* as well as the growth culture (AMS). The cultures were grown for 48-72 hours with shaking at 28°C in the AMS medium supplemented with MeOH and sodium succinate until reaching the OD_{600} of 0.8-1.0.

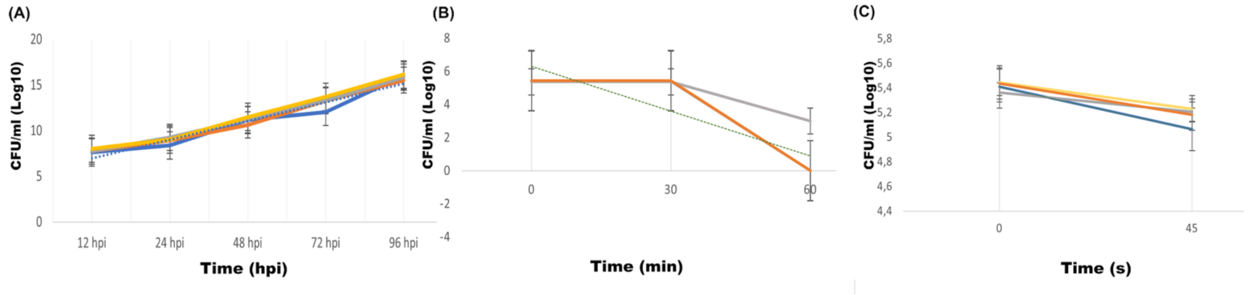

Figure S2. Growth curves of *M. extorquens* 13061 (blue) and the deletion strains *ΔphaR* (orange), *ΔphaC* (gray), and *ΔphaZ1* (yellow). (A) The strains were grown in AMS (no stress). (B) Thermal stress was applied with a 55°C heat shock. (C) UV irradiation was applied at 4.8 joules/m2 under a wavelength of 254 nm for 45 s. Growth curves were made based on the logarithmic values of CFU/ml concentrations. Sampling and calculations were done in triplicate with three technical replicates; the error bars represent standard errors.
