# Supplementary figures and images for: Biosynthesis of polyhydroxybutyrate by Methylorubrum extorquens DSM13060 is essential for intracellular colonization in plant endosymbiosis
Source: Front Plant Sci. 2024 Feb 2;15:1302705. doi: 10.3389/fpls.2024.1302705 (PMC10883064; doi:10.3389/fpls.2024.1302705)

**Baruah et al. Supplementary material: Individual CLSM images of Figure 5.**

A

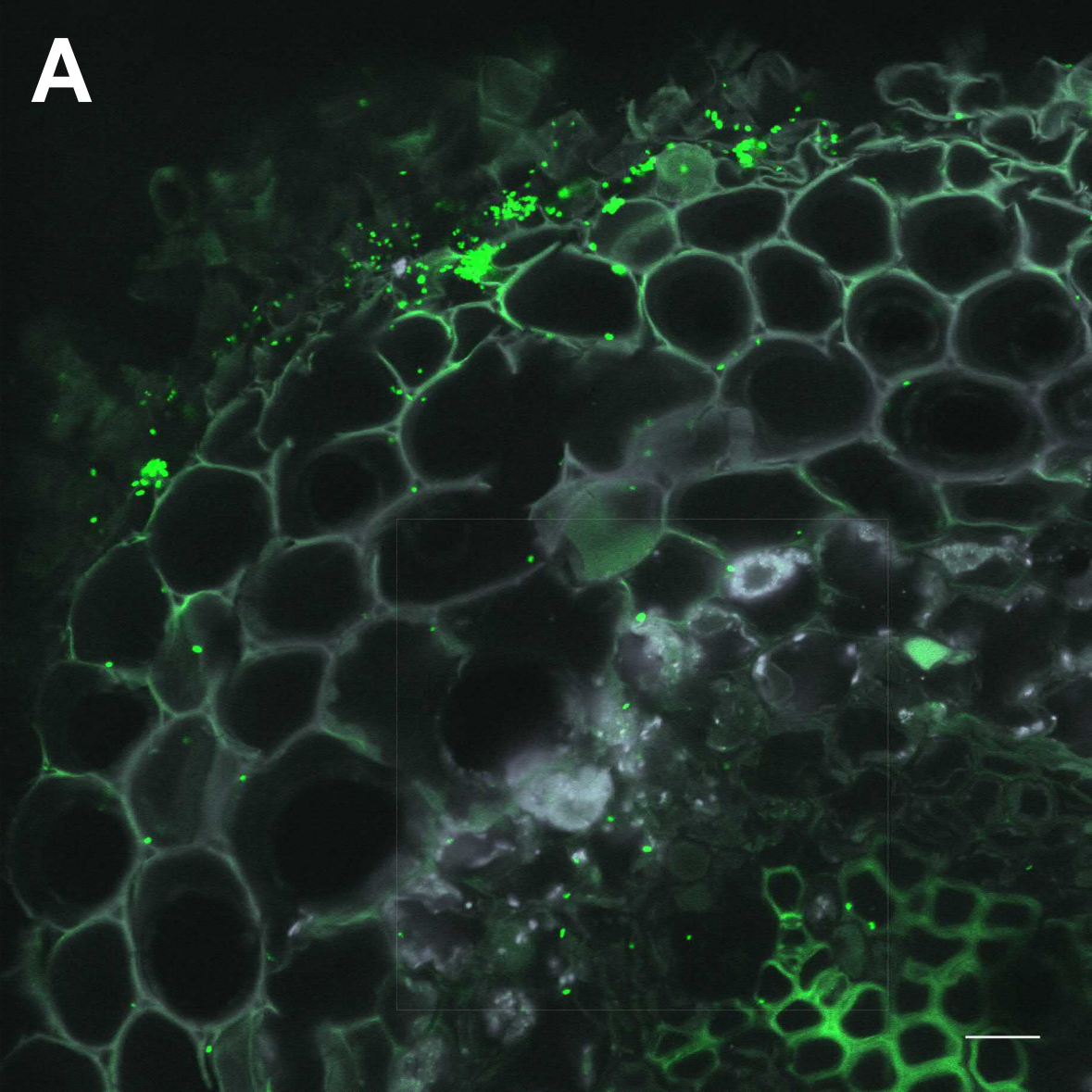

C  
A

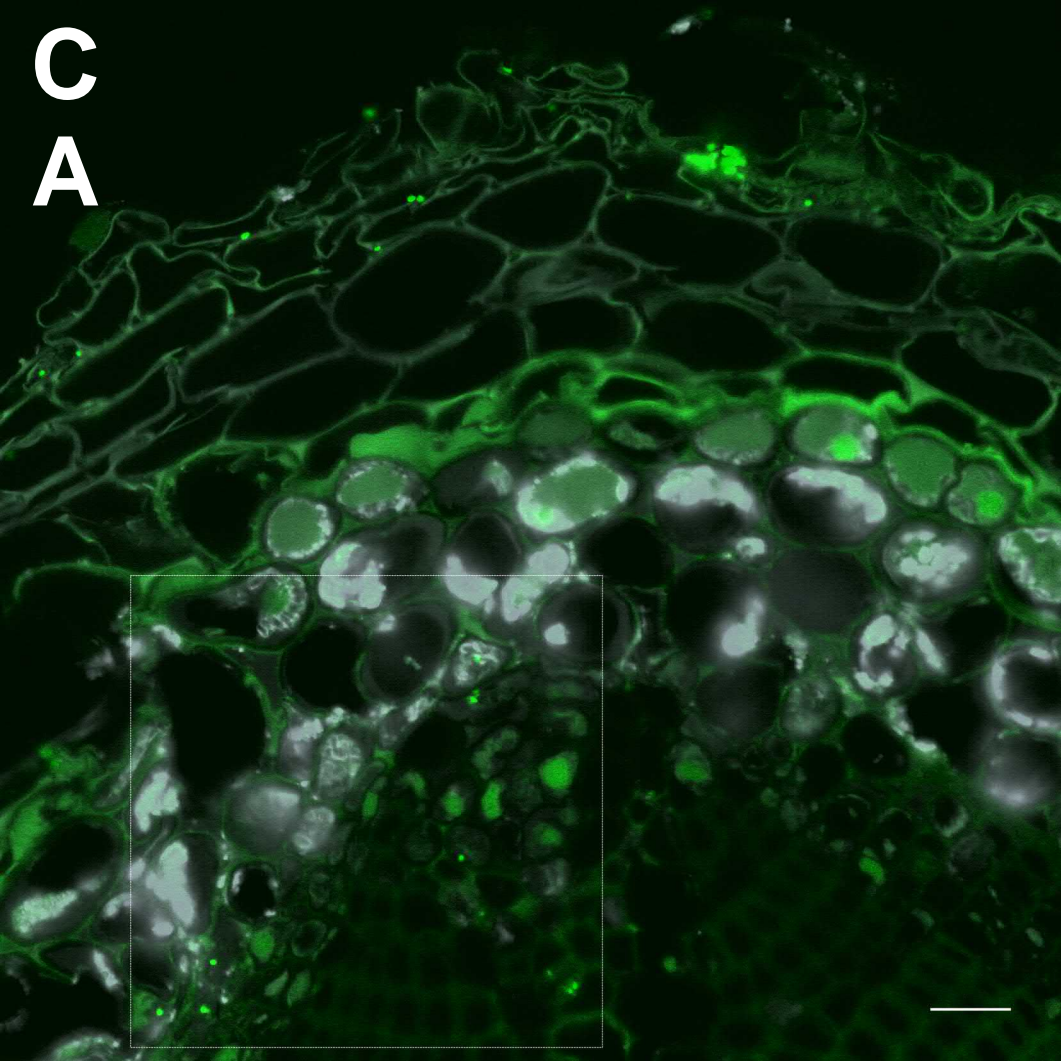

E  
A

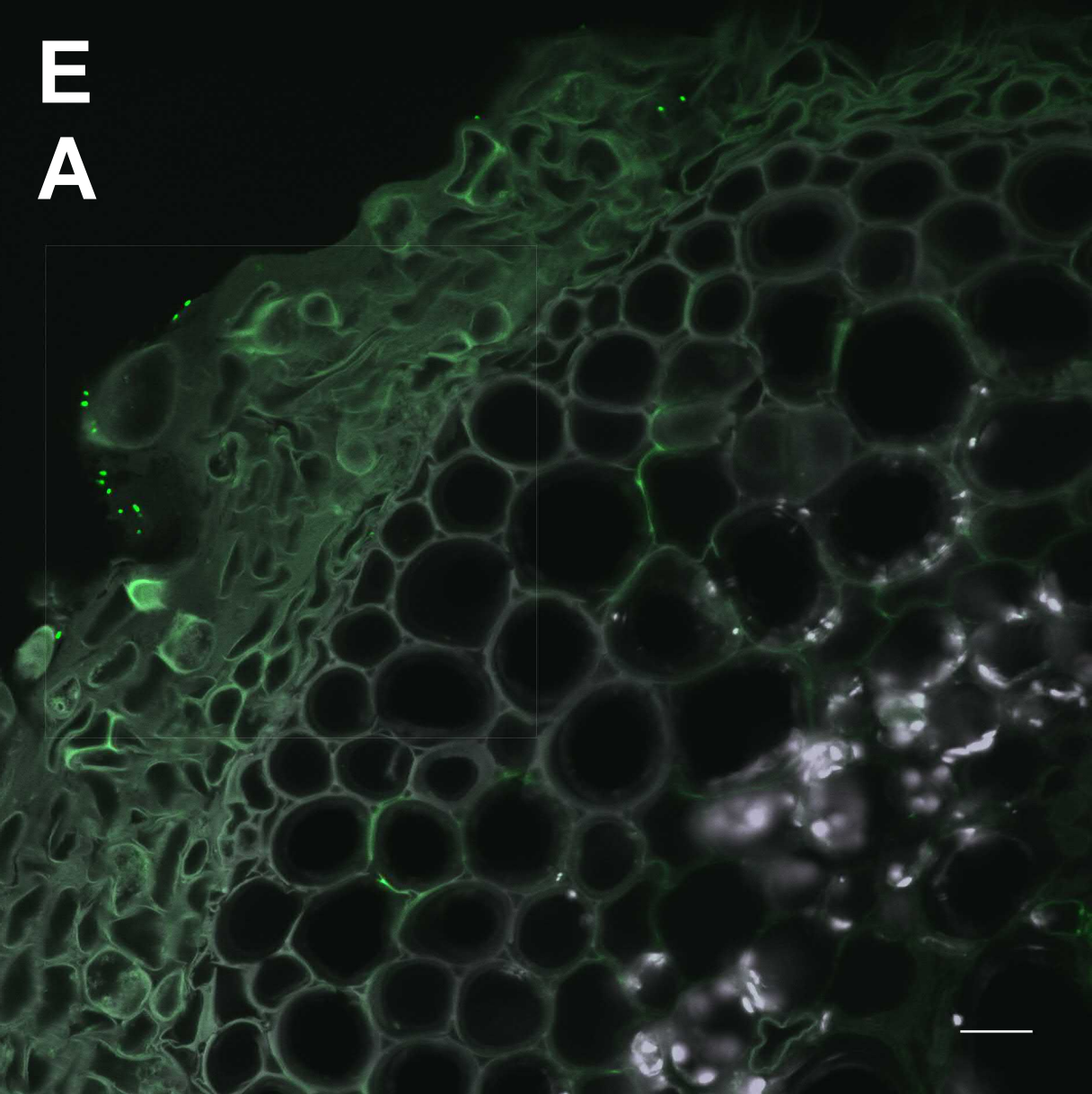

G

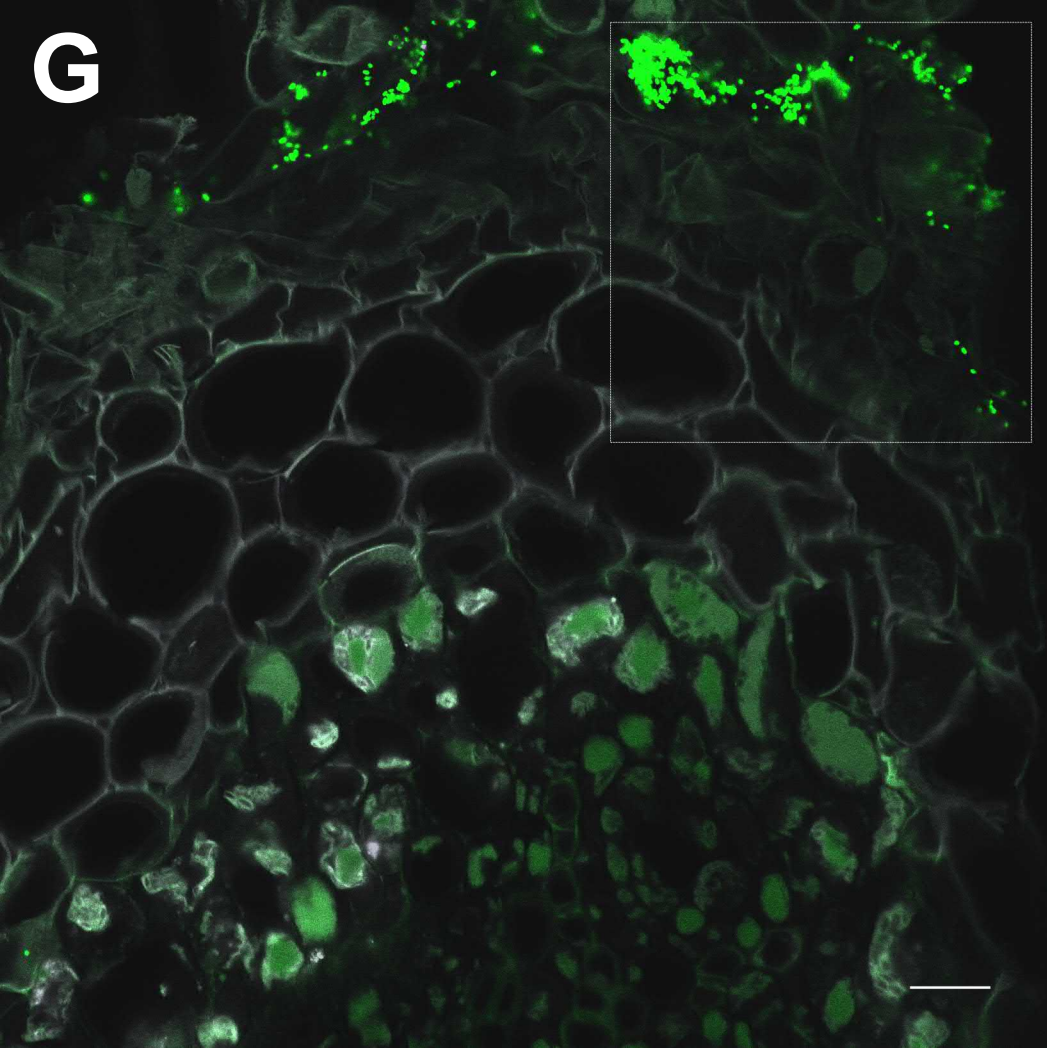

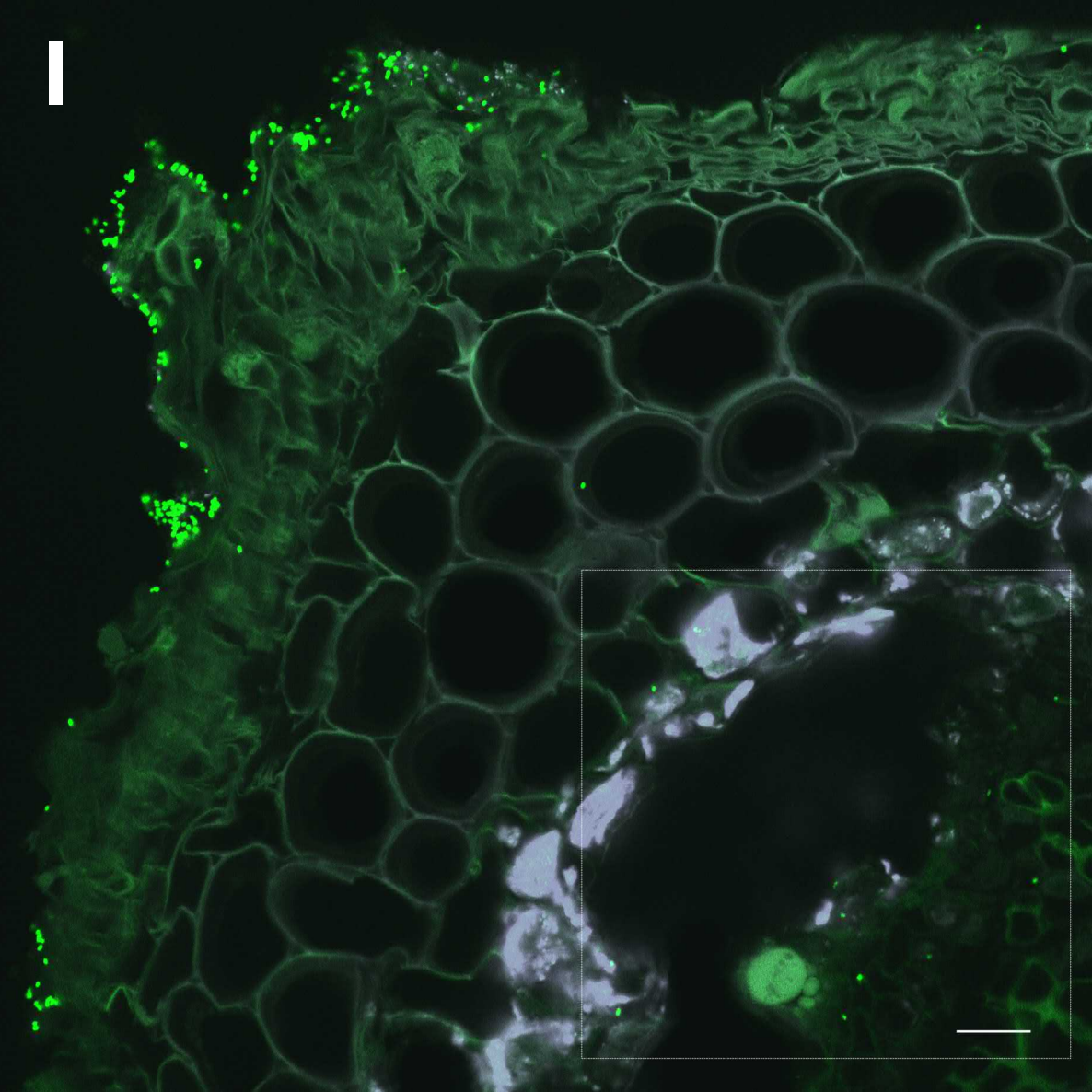

K

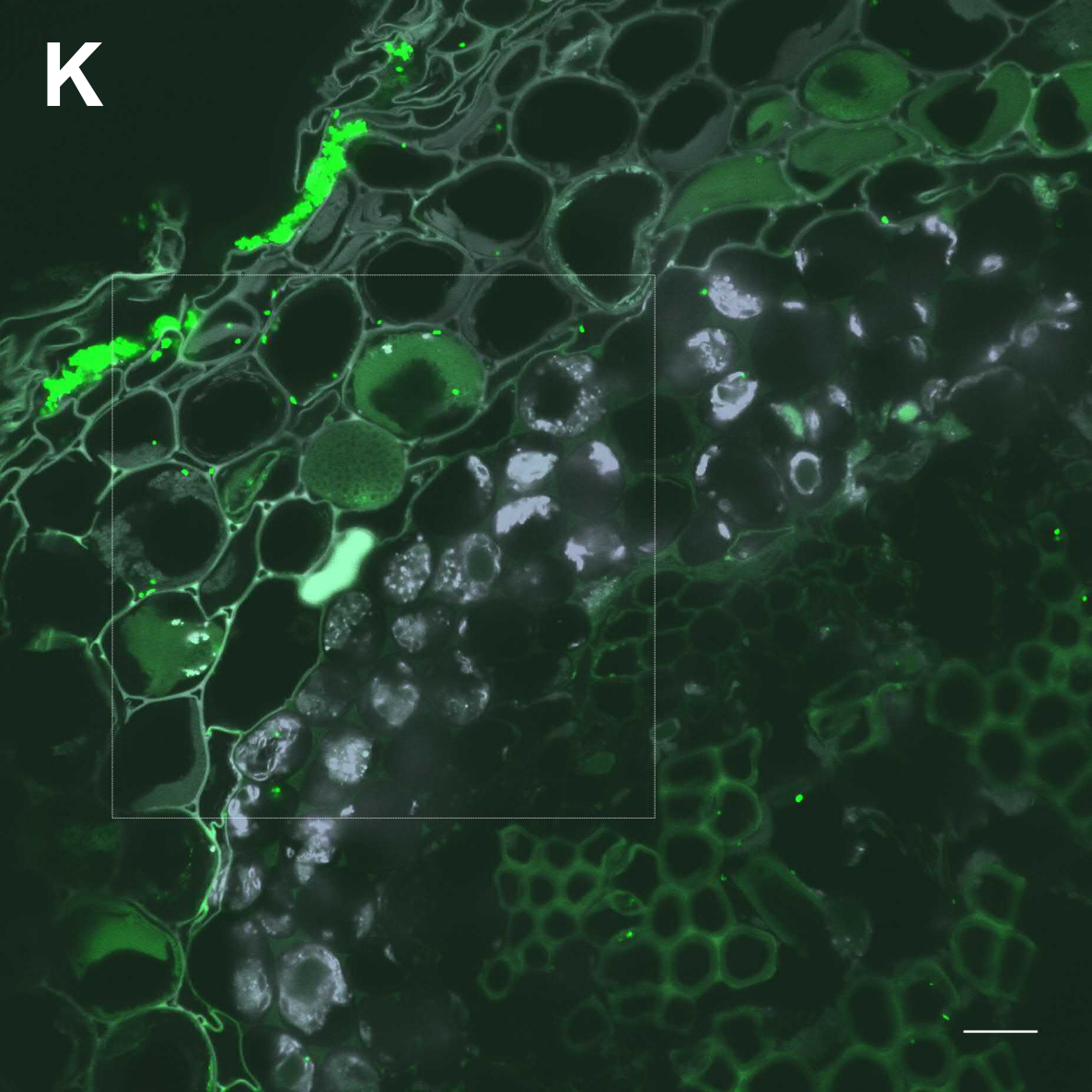

M

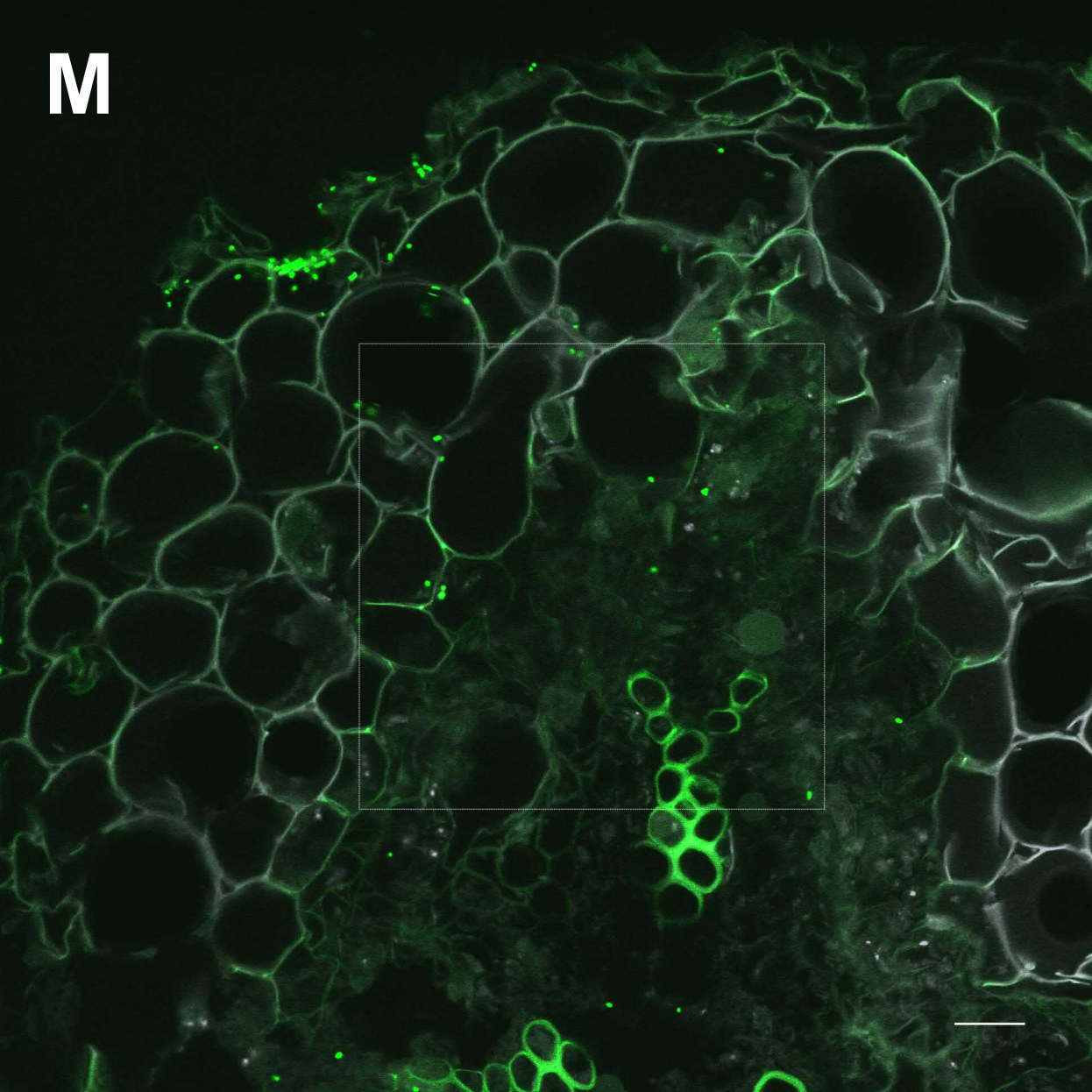

O

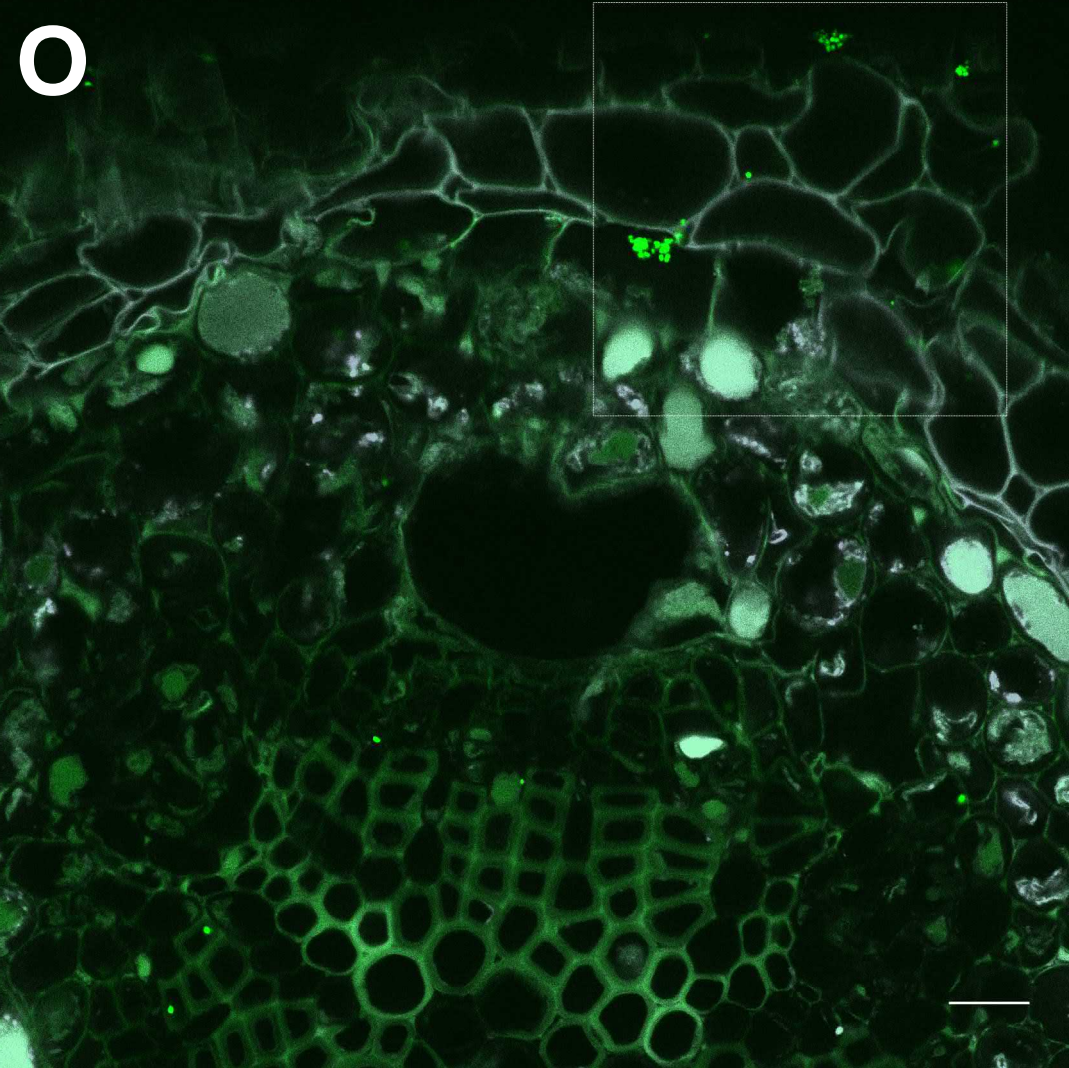

Supplement: Supplementary file 2 [file DataSheet_2.pdf]

**Baruah et al. Supplementary material: Individual CLSM images of Figure 6.**

A

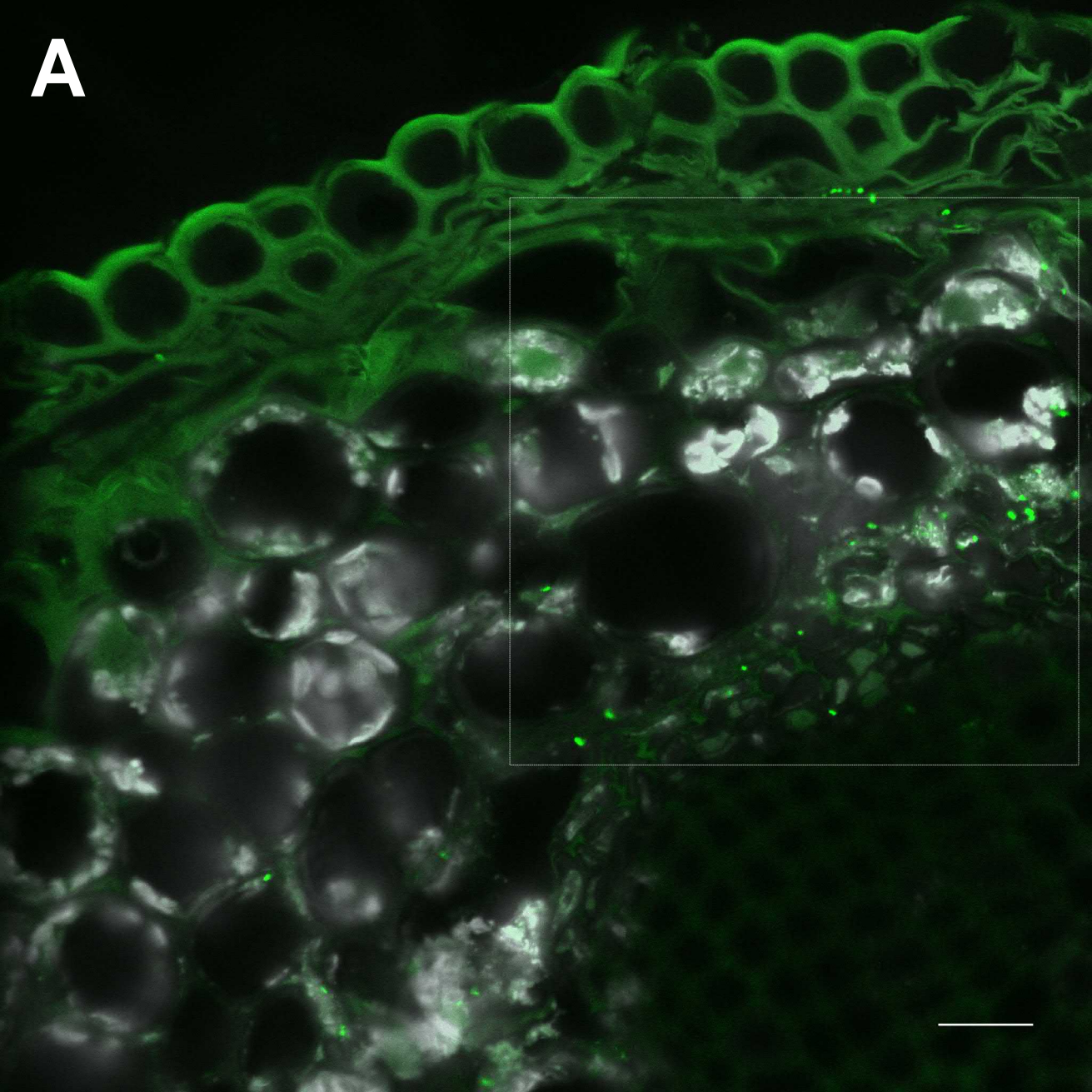

C

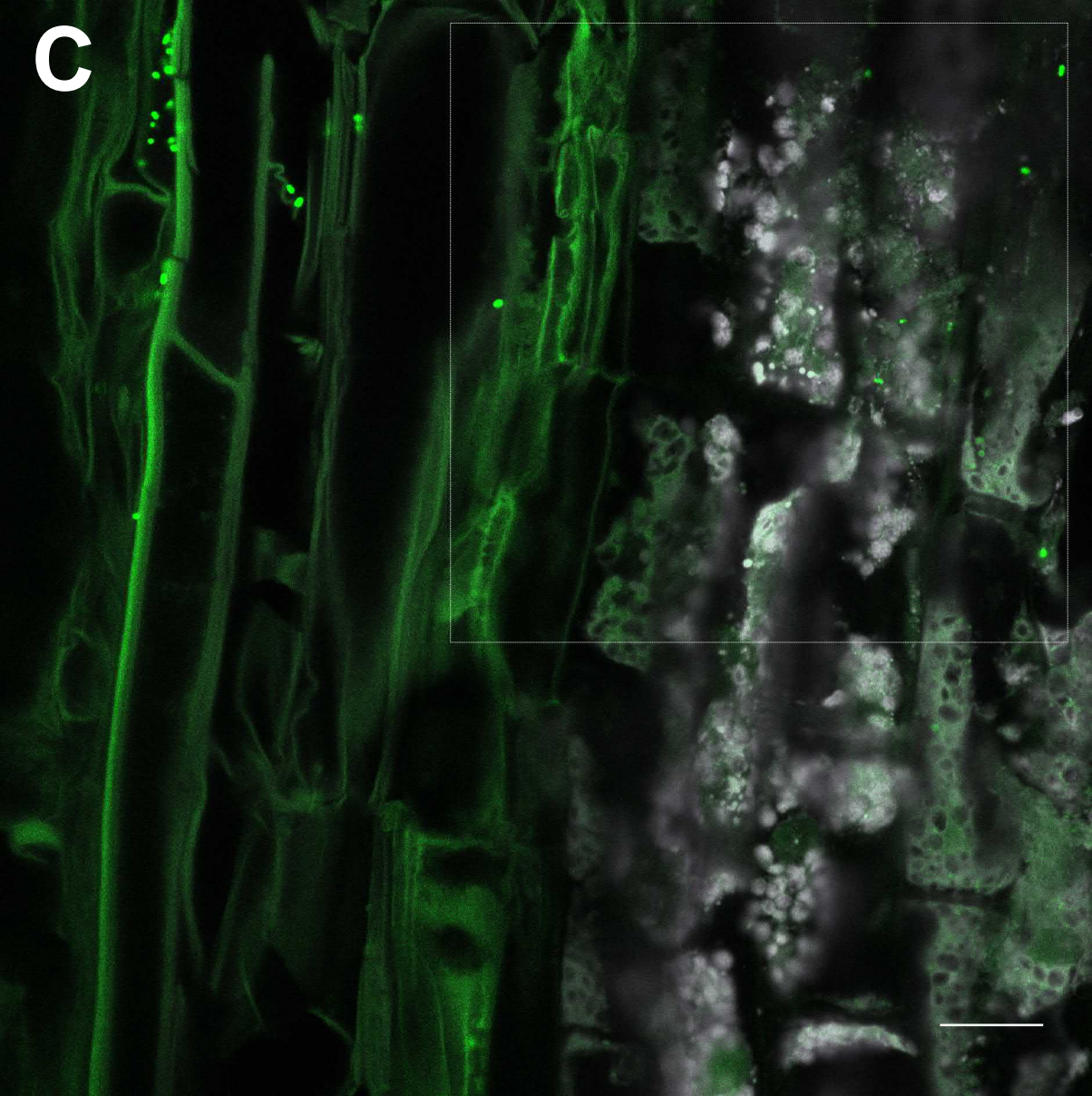

E

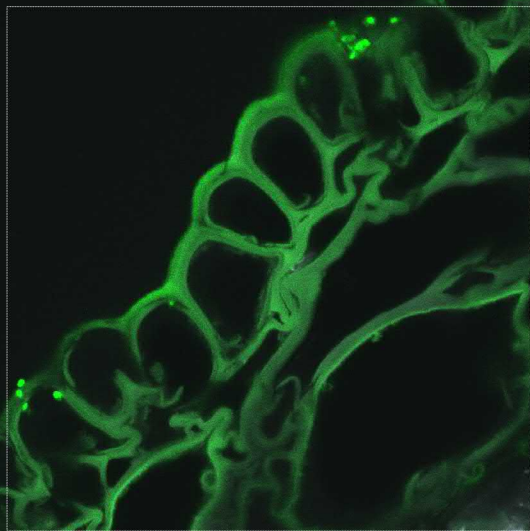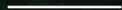

G

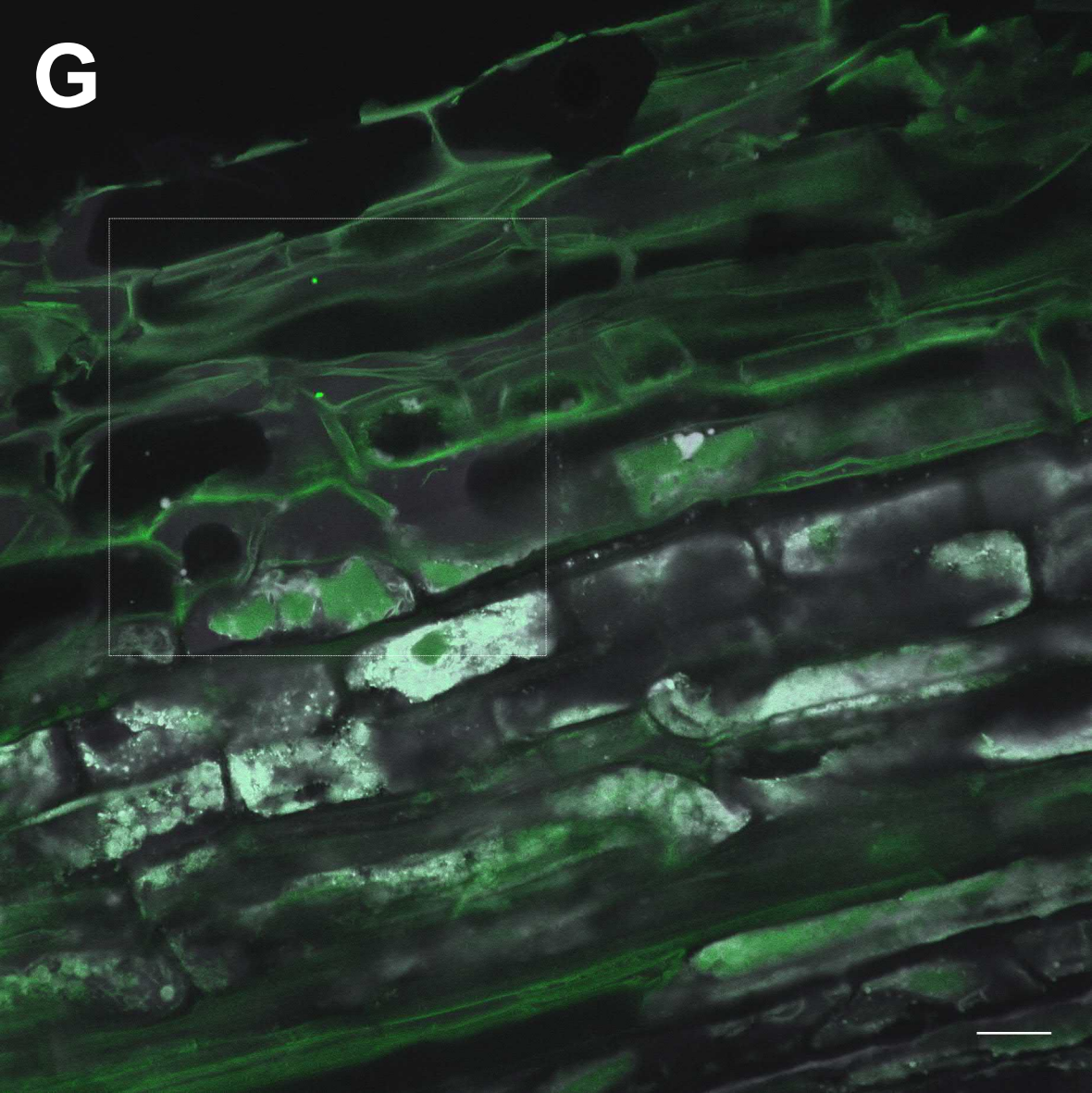

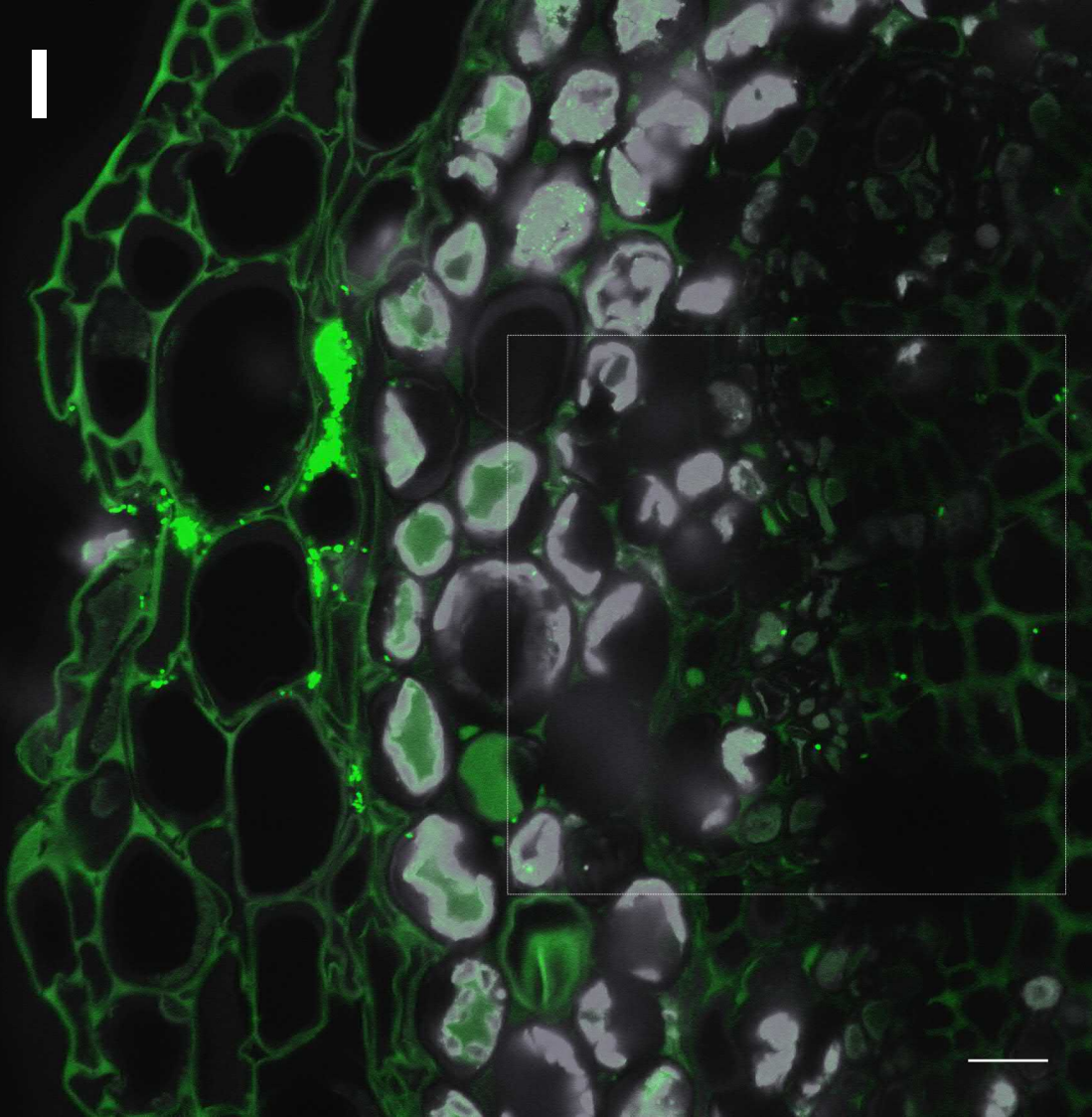

K

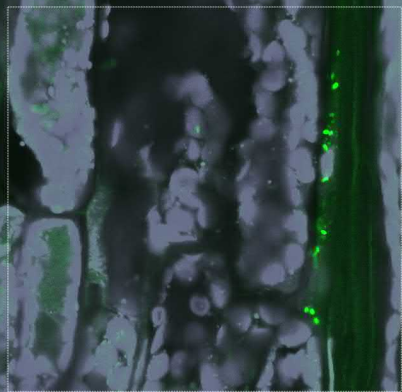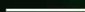

M

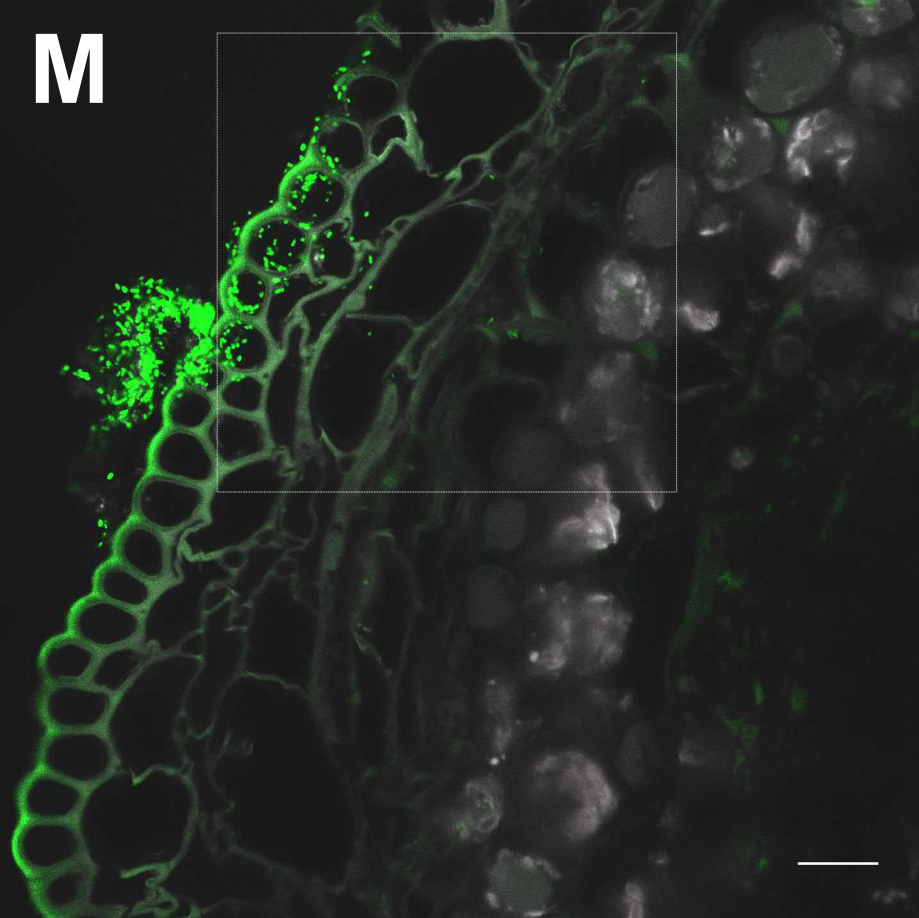

O

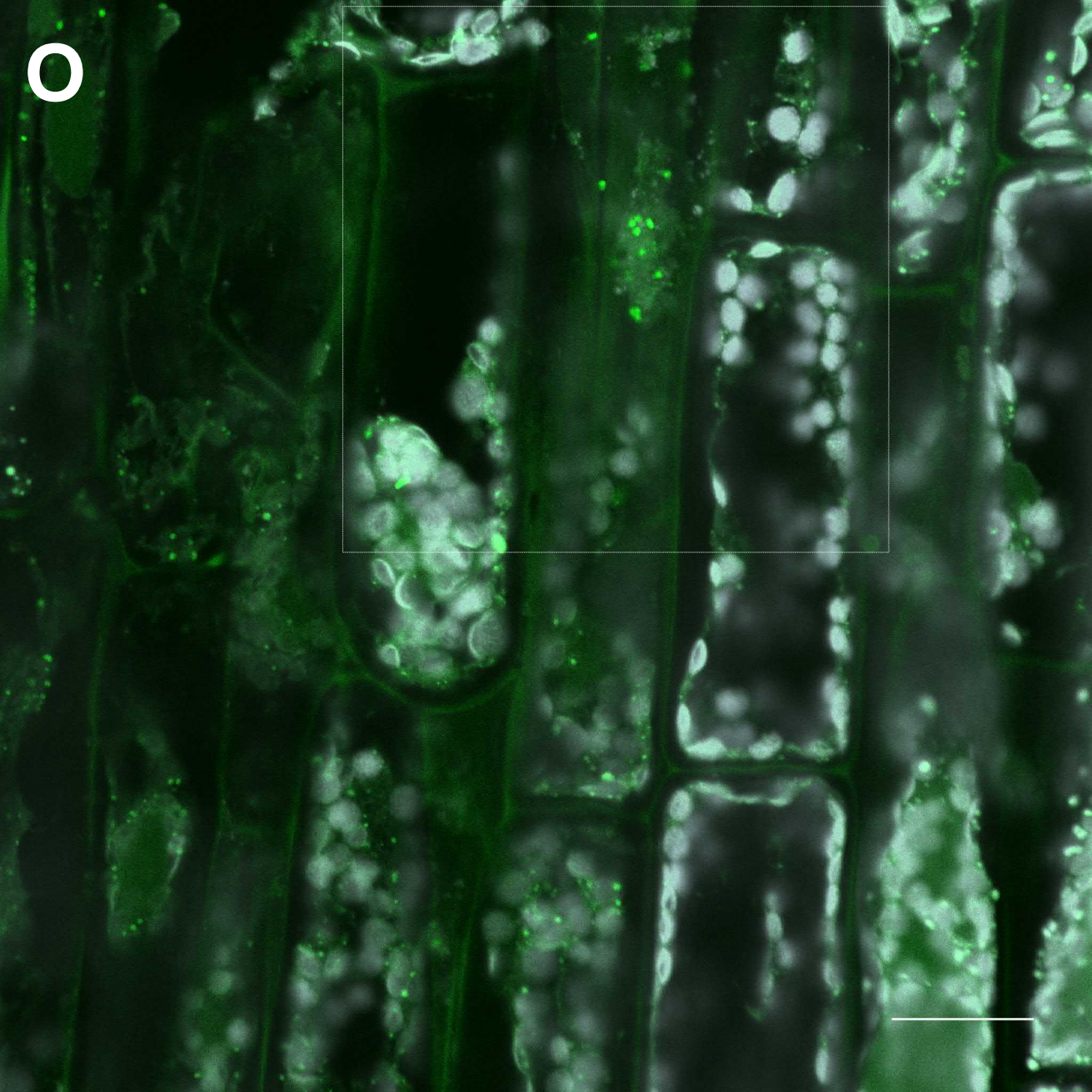

Supplement: Supplementary file 3 [file DataSheet_3.pdf]
